# A Supported Ziegler-Type Organohafnium Site Metabolizes Polypropylene

**DOI:** 10.1021/jacs.3c05940

**Published:** 2023-11-03

**Authors:** Kavyasripriya K. Samudrala, Matthew P. Conley

**Affiliations:** Department of Chemistry, University of California, Riverside, California 92521, United States

## Abstract

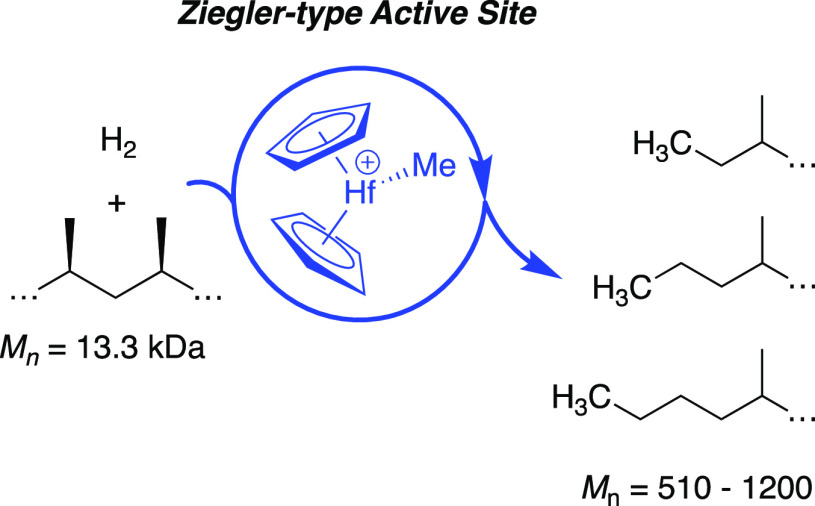

Cp_2_Hf(CH_3_)_2_ reacts with silica containing strong aluminum Lewis sites to form Cp_2_Hf–^13^CH_3_^+^ paired with aluminate anions. Solid-state NMR studies show that this reaction also forms neutral organohafnium and hafnium sites lacking methyl groups. Cp_2_Hf–^13^CH_3_^+^ reacts with isotatic polypropylene (iPP, *M*_n_ = 13.3 kDa; *Đ* = 2.4; mmmm = 94%; ∼110 C_3_H_6_/Hf) and H_2_ to form oils with moderate molecular weights (*M*_n_ = 290–1200 Da) in good yields. The aliphatic oils show characteristic ^13^C{^1^H} NMR properties consistent with complete loss of diastereoselectivity and formation of regioirregular errors under 1 atm H_2_. These results show that a Ziegler–Natta-type active site is compatible in a common reaction used to digest waste plastic into smaller aliphatic fragments.

Ziegler–Natta olefin polymerization reactions are the foundation of the plastics economy and produce millions of tons of highly versatile polyethylene or polypropylene products per year. Most polyolefins reach end-of-life as unrecyclable waste.^1^ Treating plastic waste with H_2_ and a catalyst forms low molecular weight alkanes that could be processed back to the monomer, which is a plausible strategy to a circular plastics economy. These hydrogenolysis reactions are usually catalyzed by supported nanoparticles^2^ or the heterogeneous “single-site” d^0^ metal hydrides shown in Figure 1.^3^ The well-defined catalysts activate a C–H bond in the polymer by σ-bond metathesis^4^ and β-alkyl eliminate^5^ to form MR(olefin) intermediates that are hydrogenated under the reaction conditions, Figure 1b.

**Figure 1:**
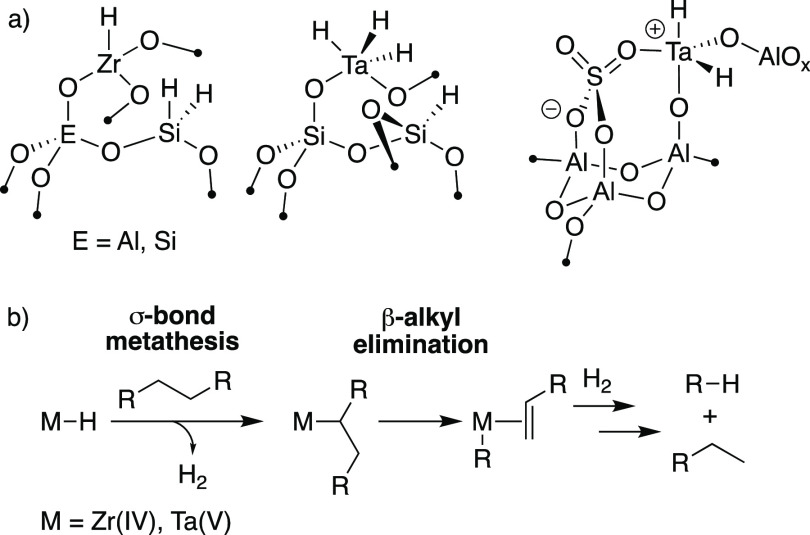
Supported d^0^ metal hydrides (a). Key steps in C–C hydrogenolysis (b).

Our hypothesis is that cationic organometallics used as olefin polymerization catalysts could engage in the reactions shown in Figure 1b. Modern olefin polymerization catalysts contain a Group IV metallocene^6^ or postmetallocene^7^ precatalyst that is activated^8^ to form L_*n*_M–R^+^ (M = Ti, Zr, Hf; R = H, alkyl) that coordinate and insert^9^ olefins to grow the polymer chain. These steps are shown in Figure 2 for the formation of isotactic polypropylene (iPP)^10^ through the common 1,2-insertion of propylene into L_*n*_M–R^+^.^11^

**Figure 2:**
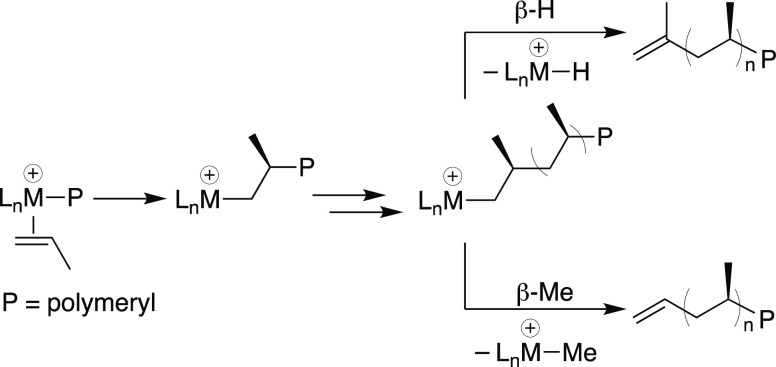
Abbreviated key steps in iPP synthesis.

Chain transfer releases the polymer from the metal to regenerate catalytically active L_*n*_M–R^+^.^12^ In the absence of alkylaluminum, the most common chain transfer pathways are β-H elimination to generate L_*n*_M–H^+^ and iPP with vinylidene end groups or β-Me elimination to generate L_*n*_M–Me^+^ and iPP with vinyl end groups. The outcome of this reaction depends on sterics. Cp_2_Hf–CH_3_^+^ terminates propylene polymerization by β-H elimination, but bulkier Cp*_2_Hf–CH_3_^+^ favors chain termination by β-methyl elimination.^13^

The β-Me elimination shown in Figure 2 is a signature of β-alkyl elimination required for C–C hydrogenolysis shown in Figure 1b. Cationic hafnocenes generated in solution are known to engage in σ-bond metathesis reactions,^14^ analogous to the d^0^ metal hydrides shown in Figure 1a.^3^ Therefore, cationic metallocenes formed during olefin polymerization reactions would be expected to show activity in reactions that digest polyolefins in the presence of H_2_. Catalysts of this type are more desirable than those shown in Figure 1. L_*n*_M–R^+^ forms readily in solution^8^ or on solid supports in common industrial compositions used for ethylene polymerization.^15^ This paper describes Cp_2_Hf–CH_3_^+^ sites on a weakly coordinating oxide^16^ that catalyze hydrogenolysis of iPP.

Silica functionalized with Al(OC(CF_3_)_3_)(PhF)^17^ forms ≡SiOAl(OC(CF_3_)_3_)_2_(O(Si≡)_2_) containing 0.21 mmol_Al_ g^–1^ and residual unreacted ≡SiOH.^18^ Cp_2_Hf(^13^CH_3_)_2_ reacts with this support to form the mixture of species shown in Figure 3a (0.21 mmol_Hf_ g^–1^). The ^13^C{^1^H} cross-polarization magic angle spinning (CPMAS) NMR spectrum of this material is shown in Figure 3b and contains signals at 38 (Hf–^13^CH_3_^+^), 24 (Hf–^13^CH_3_), 2 (Si–^13^CH_3_), and −11 ppm (Al–^13^CH_3_), respectively. The reactivity shown in Figure 3a can be rationalized by the following chemical steps. [Cp_2_Hf–^13^CH_3_][≡SiOAl(OC(CF_3_)_3_)_2_(CH_3_)] (**1**) forms by methide abstraction from Cp_2_Hf(^13^CH_3_)_2_ by the strong aluminum Lewis sites, analogous to reactions of B(C_6_F_5_)_3_ with d^0^ organometallics in solution.^19^ Residual −OH sites present on the support react with Cp_2_Hf(^13^CH_3_)_2_ to form CH_4_ (0.07 ± 0.01 mmol_CH_4__ g^–1^) and Cp_2_Hf(^13^CH_3_)(OSi≡) (**2**). This result indicates that ∼30% of the Lewis sites in ≡SiOAl(OC(CF_3_)_3_)_2_(O(Si≡)_2_) do not react with Cp_2_Hf(^13^CH_3_)_2_. **3** forms when Hf–Me^+^ in **1** reacts with a nearby siloxane bridge to generate [Cp_2_Hf(OSi≡)][≡SiOAl(OC(CF_3_)_3_)_2_(CH_3_)] and ≡Si–^13^CH_3_, which is also observed when Cp_2_Zr–CH_3_^+^ fragments are generated on silica.^20^

**Figure 3:**
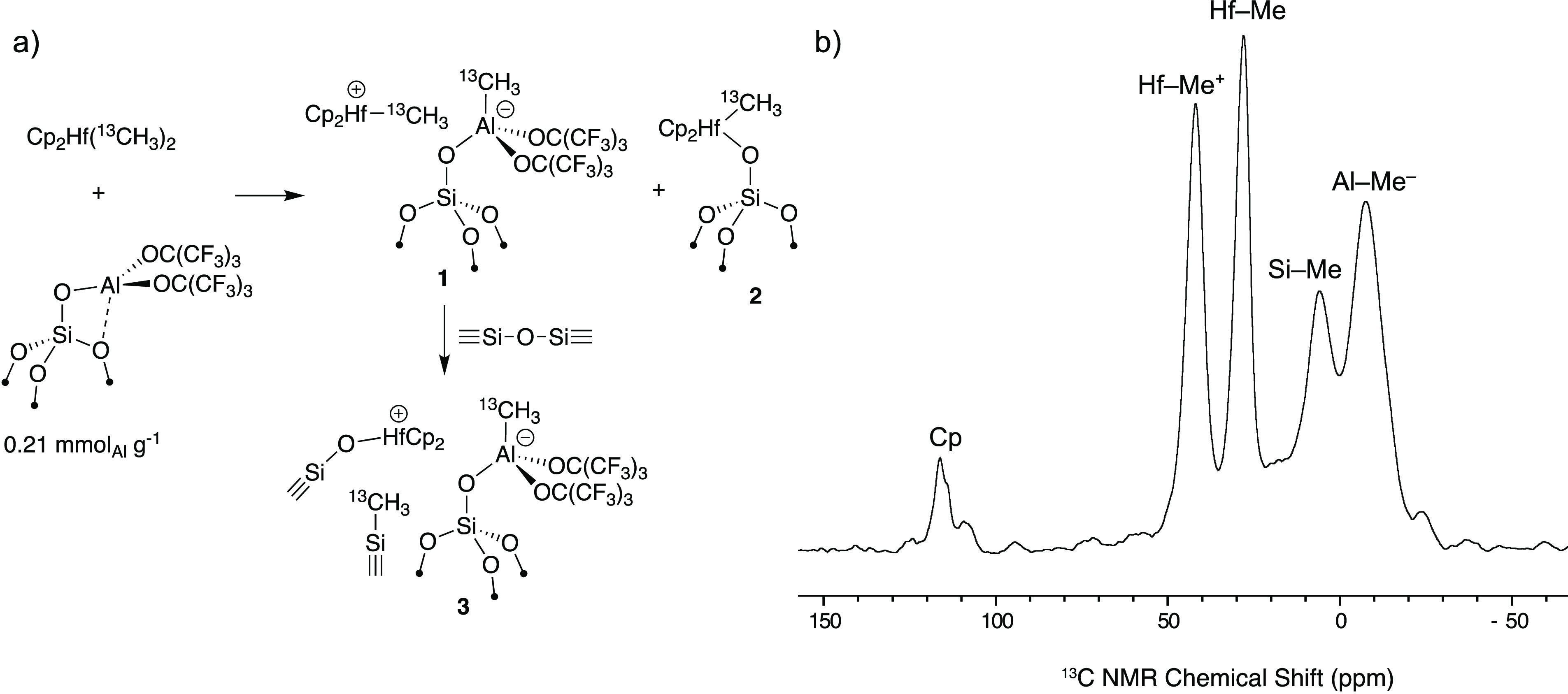
Reaction of Cp_2_Hf(CH_3_)_2_ and ≡SiOAl(OC(CF_3_)_3_)_2_(O(Si≡)_2_) to form **1**, **2**, and **3** (a). ^13^C{^1^H} CPMAS NMR spectrum of the reaction products (b). ν_rot_ = 10 kHz.

The catalytic properties of Cp_2_Hf(CH_3_)_2_/≡SiOAl(OC(CF_3_)_3_)_2_(O(Si≡)_2_) in a melt of iPP (*M*_n_ = 13.3 kDa; *Đ* = 2.4; mmmm = 94%; ∼110 C_3_H_6_/Hf) with H_2_ are given in Table 1. These reactions form a complex mixture of saturated alkane products lacking diastereopurity, as shown in eq 1. Similar to previous studies,^3g^ high temperature ^13^C{^1^H} NMR analysis of the recovered unreacted polymer maintains high mmmm purity.
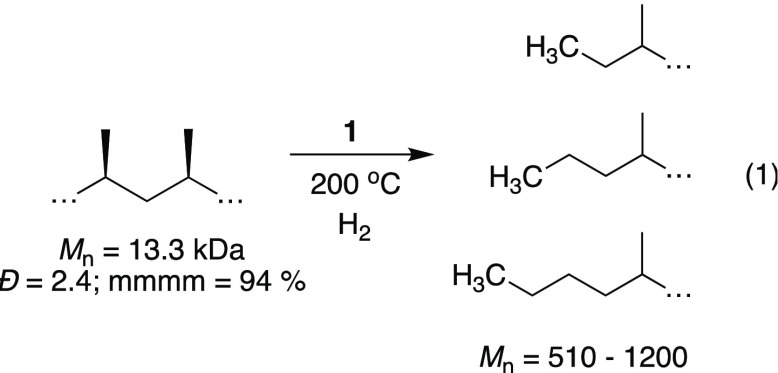
1

**Table 1:** Catalytic Activity of **1** in PP Hydronenolysis^a^

Entry	Pressure (atm)	Yield C_1_–C_5_ (%)	Yield Oil^b^ (%)	*M*_n_^c^ (g/mol)
1	1^d^	2	62	390
2	1^e^	12	83	290
3^f^	1^e^	7	38	350
4	5	4	62	220
5	10	8	88	240
6	2^g^	n.d.	29	1100
7	5^g^	n.d.	20	1200

a Reactions run with 200 mg of iPP and 200 mg of Cp_2_Hf(CH_3_)_2_/≡SiOAl(OC(CF_3_)_3_)_2_(O(Si≡)_2_) (0.042 mmol of Hf) at 200 °C for 24 h at the pressure given in the table.

b (mg_oil_)/(mg_iPP_).

c Determined from quantitative ^13^C{^1^H} NMR of extracted oils.

d H_2_:Hf ∼ 100.

e H_2_:Hf (or Zr) ∼ 1500.

f Zr derivative of **1**, reported in ref (17).

g Performed with H_2_ fed to the reactor on demand (see the Supporting Information for details). n.d. = not determined.

Available data suggest that a Hf–CH_3_^+^ is the catalytically active site in Cp_2_Hf(CH_3_)_2_/≡SiOAl(OC(CF_3_)_3_)_2_(O(Si≡)_2_. Native ≡SiOAl(OC(CF_3_)_3_)_2_(O(Si≡)_2_) sluggishly converts iPP to extractable oils in the presence of 1 atm H_2_ (15% yield), suggesting that unreactive Lewis sites play a minimal role in the catalytic chemistry involving **1**. Reacting Cp_2_Hf(CH_3_)_2_/≡SiOAl(OC(CF_3_)_3_)_2_(O(Si≡)_2_ with H_2_ at 150 °C for 12 h in the absence of iPP forms methane (0.09 mmol g^–1^), and the FTIR of this material contains a broad signal at 1650 cm^–1^ tentatively assigned to a hafnium hydride. This reactivity pattern is expected from extensive precedent in the homogeneous and heterogeneous literature showing that M–R species react with H_2_ to form M–H and RH.^3a^ Under identical conditions, independently synthesized **2** forms only 0.001 mmol_CH_4__ g^–1^. In addition, **2** does not react with iPP under hydrogenolysis conditions to form extractable oils nor incorporate deuterium into residual iPP in the presence of D_2_ (see the Supporting Informaiton for details).

After 24 h at 200 °C under 1 atm H_2_, **1** forms an oil in 62% yield, Entry 1. Analysis of the gas phase before extraction of the oil shows that only 2% of the polymer is converted to light gases (1.5 CH_4_ Hf^1–^, 0.01 C_2_H_6_ Hf^1–^, 0.11 C_3_H_6_ Hf^1–^, and 1.1 C_4_H_10_ Hf^1–^, 1.2 C_5_H_10_ Hf^1–^). Increasing the H_2_:Hf ratio to ∼1500 results in near complete conversion of iPP to oil (83%) and light gas (12%), Entry 2. The zirconium derivative of **1**(18) is also active in this reaction but produces less oil (38%) and light gas (7%) than hafnium, Entry 3. A closed Parr reactor charged with 5 or 10 atm H_2_ also results in a good yield of oils with minimal volatile gas formation (Entries 4 and 5). However, at 2 or 5 atm with H_2_ supplied on demand, conditions that prevent recovery of volatile gases, yields of extracted oils drop (Table 1, Entries 6 and 7). The ^1^H NMR data for the extracted oils are largely uninformative, but these spectra contain signals for internal olefins ranging from ∼1:50 to ∼1:2000 olefin:C_3_H_6_ unit, depending on the conditions (see the Supporting Information).

Quantitative ^13^C{^1^H} NMR spectra of the oils in C_6_D_6_ are shown in Figure 4. Most spectra in Figure 4 contain signals that are characteristic of regioirregular errors encountered in polypropylene synthesis,^11^ or copolymerization reactions of ethylene and propylene.^21^ These results indicate that some degree of chain straightening occurs during hydrogenolysis with **1**. All ^13^C{^1^H} NMR spectra contain signals for ethyl, propyl, and butyl end groups. Integration of the end groups relative to the rest of the ^13^C{^1^H} NMR signals gives the *M*_*n*_ values of the oils reported in Table 1. The matrix assisted laser desorption ionization (MALDI) mass spectrum of extracted oil from Entry 1 contains a broad distribution of products centered at a *m*/*z* of 538 (∼10 C_3_H_6_ units *Ag^+^) that is close to *M*_*n*_ obtained from integration of ^13^C{^1^H} NMR signals. GCMS data of the oil is complex but also supports the formation of a distribution of branched alkanes. These trends hold for all oils isolated in Table 1.

**Figure 4:**
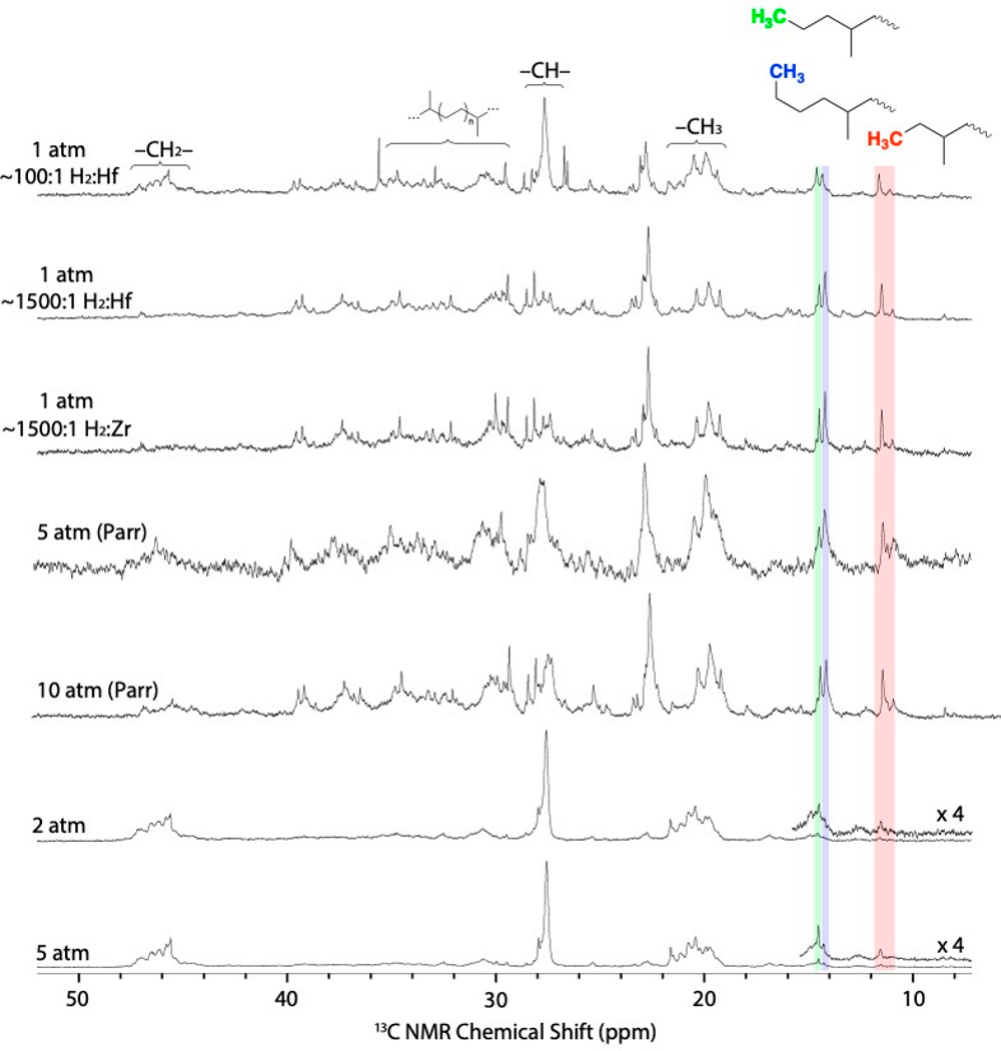
Quantitative ^13^C{^1^H} NMR spectra shown from 10 to 50 ppm for oils produced in hydrogenolysis reactions. End groups are highlighted for clarity.

Reactions of iPP with **1** and D_2_ (1 atm, D_2_:Hf ∼ 100) at 200 °C also result in formation of oils and small amounts of light gas in similar yields as those performed with H_2_. The ^2^H NMR spectrum of unreacted iPP at 120 °C in C_2_H_2_Cl_4_ contains signals for −CD–, −CHD–, and −CH_*x*_D_3–*x*_ in a ∼1:1:2 ratio, eq 2. This spectrum also contains signals for CH_3_CH_2_CD_2_CD=C(CH_3_)P (P = polymeryl). The oils formed in this reaction also contained deuterium at all possible positions (−CD–:–CHD–:–CH_*x*_D_3–*x*_ ∼ 1:4:10).
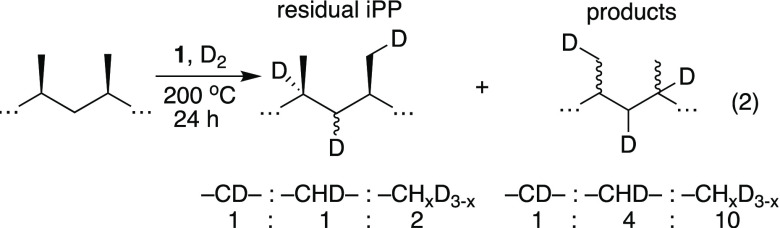
2

These data suggest that Hf–H^+^ reacts with a primary or secondary C–H bond in iPP by σ-bond metathesis to form Hf–R^+^, Scheme 1.^22^ β-Alkyl elimination forms Hf(R)(olefin)^+^ intermediates that are hydrogenated by H_2_. Hf(R)(olefin)^+^ probably dissociates olefin to allow the hydrogenolysis of Hf–R^+^ by a σ-bond metathesis reaction prior to olefin hydrogenation. This process forms propyl (observed) and isopropyl (not observed) end groups. Deuterium incorporation into recovered iPP suggests that the σ-bond metathesis reactions shown in Scheme 1 are reversible and accounts for the −CHD– and −CD_*x*_H_3–*x*_ in recovered iPP and atactic oils.

**Scheme 1:**
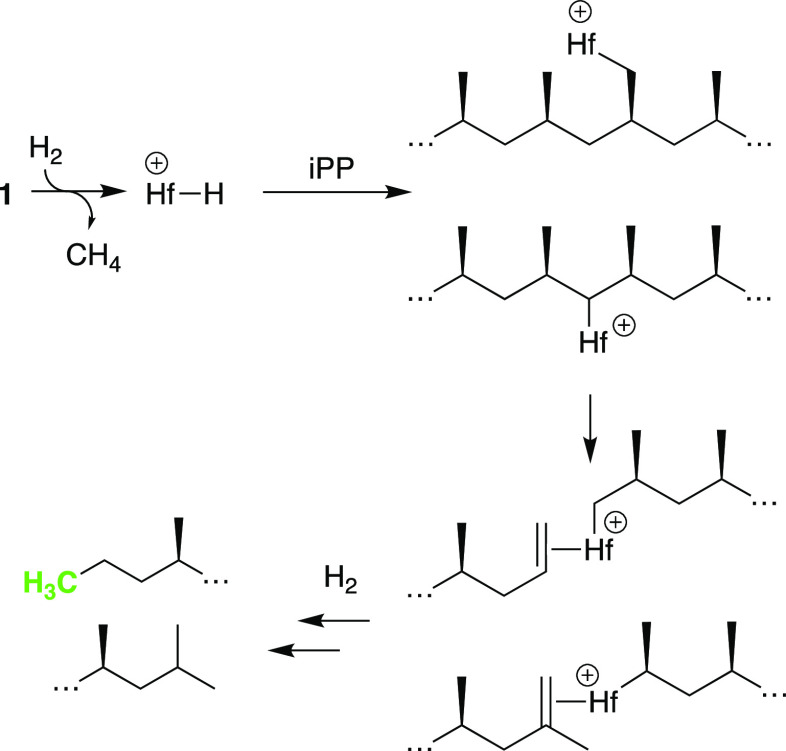
Cleavage of an iPP Chain by **1**

The atactic oils must lose diastereoselectivity. Epimerization can occur by β-H elimination, nondissociative alkene flipping,^23^ and unselective olefin insertion,^24^ shown in Scheme 2. This process incorporates deuterium at the −CD– position in the oils or iPP. Deuteration of −CH– positions can also occur by reactions of 3° Hf–R^+^ with D_2_. Residual Lewis acidic aluminum in Cp_2_Hf(CH_3_)_2_/≡SiOAl(OC(CF_3_)_3_)_2_(O(Si≡)_2_) could also promote isomerization reactions, resulting in loss of tacticity.^25^ Why the oils are atactic and the recovered iPP maintains high isotacticity is currently unclear but could imply that unreacted iPP chains are the dominant species in the recovered polymer.

**Scheme 2:**
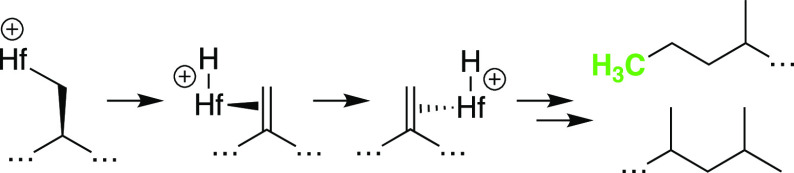
Epimerization of a Methyl Group Mediated by Hf–H^+^

The atactic oils also incorporate regioirregular “errors” into the chain. Quantitative ^13^C{^1^H} NMR data of extracted oils suggest that regioirregular errors occur throughout the alkyl chain and not solely at chain ends. In propylene polymerization reactions the regioirregular errors, formally 3,1 insertion products, arise from 1,2-insertion of propylene to form a 2° M–R that eliminates β-H and reinserts to form a 1° M–R, Figure 5a.^11^ This pathway is not plausible under the hydrogenolysis conditions. Instead, C–H bond activation by Hf–H^+^ generates a Hf–R^+^ that undergoes β-alkyl elimination, and 2,1-reinsertion gives the chain-straightened product after hydrogenolysis, Figure 5b. Reactions from a terminal isopropyl position generate the butyl end group. If the chain-straightened intermediate β-alkyl eliminates, the ethyl end group forms after hydrogenation.

**Figure 5:**
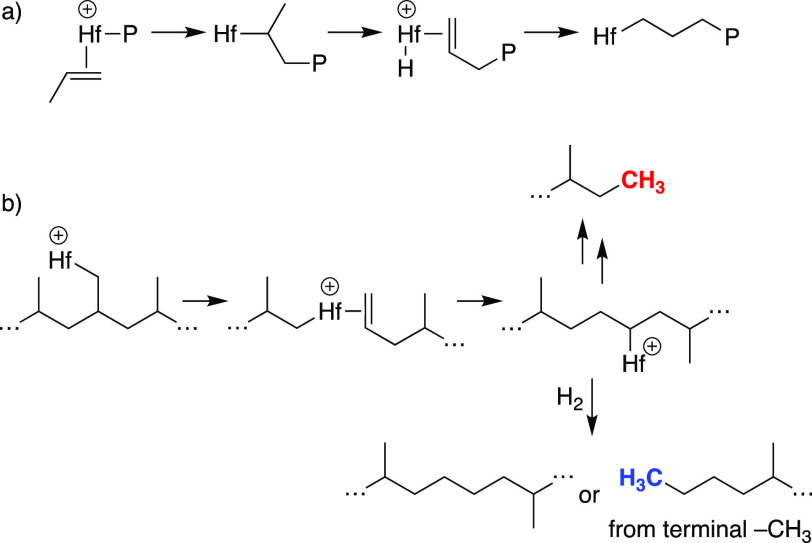
Steps involved in a formal 3,1-insertion of propylene during polymerization reactions (a). Regioirregular error formation under hydrogenolysis conditions (b).

Until this report the most common catalysts for hydrogenolysis of polyolefins were supported noble metal nanoparticles^2^ or supported d^0^ metal hydrides (Figure 1a).^3^ Comparisons between these disparate classes of catalysts are difficult, but **1** does appear to offer some advantage. **1** selectively produces long chain hydrocarbons and avoids significant formation of light gases at prolonged reaction times. We suspect that the selectivity rests on the moderate activity of **1** in the reactions involved in hydrogenolysis of iPP. For example, under essentially identical reaction conditions Ta–H^+^ sites supported on sulfated aluminum oxide converts the same iPP to shorter liquid hydrocarbon fragments and more light gas, indicating that Ta–H^+^ facilitates more chain cleavage than **1**.^3g^

Metallocene catalysts that polymerize olefins have been overlooked as catalysts for degradation of aliphatic polyolefin plastics.^26^ The reactivity of **1**, and the inactivity of **2**, in iPP hydrogenolysis show the critical role of forming an organometallic ion-pair in this reaction. Many modern Ziegler–Natta catalysts for olefin polymerization contain mixtures of metallocene, aluminum alkyl (or methaluminoxane), and an oxide support; and these mixtures likely self-assemble to form ion pairs similar to the Hf–H^+^ derived from **1**.^15^ A plausible implication of the results shown here is that the broad portfolio of modern Ziegler–Natta catalysts available for olefin polymerization may also catalyze reactions that degrade the polymers that these catalysts produce.

## Supporting Information Available

Experimental methods, FT-IR spectra, solid-state NMR spectra, gas chromatograph traces, MALDI MS, GCMS, and quantitative ^13^C{^1^H} NMR spectra of extracted oils (PDF)
