# Supplementary material for: A Supported Ziegler-Type Organohafnium Site Metabolizes Polypropylene
Source: J Am Chem Soc. 2023 Nov 3;145(45):24447–51. doi: 10.1021/jacs.3c05940 (PMC10655186; doi:10.1021/jacs.3c05940)
Supplement: Supplementary file 1 — ja3c05940_si_001.pdf [file ja3c05940_si_001.pdf]

Supplementary Materials for:

A Supported Ziegler-Type Organohafnium Site Metabolizes Polypropylene

Kavyasripriya K. Samudrala and Matthew P. Conley*
Department of Chemistry, University of California, Riverside, California 92521, United States

Table of contents

1. General considerations S2
2. Synthesis and Characterization of $Cp_2Hf(CH_3)_2$/≡SiOAl($OC(CF_3)_3)_2$(O(Si≡$)_2$) S3
3. Synthesis and Characterization of $Cp_2Hf(^{13}CH_3)$(OSi≡) (**2**) S5
4. Reaction of Cp_2HfMe_2/≡SiOAl($OC(CF_3)_3)_2$(O(Si≡$)_2$) or $Cp_2Hf(CH_3)$(OSi≡) with H_2. S8
5. Procedure for hydrogenolysis of iPP S9
6. GC analysis of volatile products S11
7. MALDI MS data of extracted oils S14
8. GCMS of oils from hydrogenolysis of iPP S19
9. ^1H NMR data for extracted oils S23
10. Solution ^2H NMR data for extracted oil and residual iPP S25
11. Representative solution ^1H and $^{13}C\{^1H\}$ NMR data for residual iPP S26
12. Integral table for M_n quantification of extracted oils S27
13. iPP degradation reactions using ≡SiOAl($OC(CF_3)_3)_2$(O(Si≡$)_2$). S28
14. Lack of reactivity of iPP with $Cp_2Hf(CH_3)$(OSi≡) (2). S31

General Considerations

All manipulations were performed under an inert atmosphere of dinitrogen or argon using standard Schlenk or glovebox techniques. C_6D_6 was purchased from Cambridge Isotope Laboratories, dried over sodium/benzophenone, degassed by three successive freeze-pump-thaw cycles, distilled under vacuum, and stored in an inert atmosphere glovebox. $C_2D_2Cl_4$ was purchased from Cambridge Isotope Laboratories and used as received. Pentane was dried by passing through a J.C. Meyer solvent system containing two activated alumina columns, stored over sodium/benzophenone, degassed, and distilled under vacuum. Hydrogen (UHP grade) was purchased from Airgas and was passed through oxygen/water trap (CRS, ZPure H_2O/O_2) immediately before use. Isotactic polypropylene (M_n =13.3 kDa) was purchased from Sigma-Aldrich and used without further purification. Deuterium was purchased from CIL and was dried/deoxygenated using activated 4Å molecular sieves and regenerated BASF Cu catalyst. $\equiv SiOAl(OC(CF_3)_3)_2(O(Si\equiv)_2)$ was prepared as previously described.[1] Cp_2HfMe_2 and $Cp_2Hf(^{13}CH_3)_2$ were prepared as reported.[2] FTIR spectra were recorded in transmission mode as pressed pellets using a Bruker Alpha IR spectrometer in an argon-filled glovebox. Elemental analysis of Al and Hf were carried out by digesting solid samples in 2% nitric acid for 12 hours at room temperature and measuring samples at the University of California, Riverside Environmental Sciences Research Laboratory (ESRL) on a Perkin-Elmer Optima 7300DV ICP-OES.

Solution NMR data (^1H, ^2H, and $^{13}C\{^1H\}$) was acquired at 14.1T on an Avance Bruker 600 MHz NMR spectrometer. ^1H and ^{13}C NMR spectra were referenced to the residual proton signal from the NMR solvent. Quantitative $^{13}C\{^1H\}$ NMR experiments were acquired using an inverse-gated decoupling pulse sequence using a 90° pulse of 9.0 µs, a relaxation time of 5 s and an acquisition time of 2 s. Samples for this measurement were prepared at 10% weight solution of polymers in 0.05 M $Cr(acac)_3$ dissolved in 1,1,2,2-tetrachloroethane-d_2 solution at 120 °C. Analogous procedures were used to analyze oils in C_6D_6 solution at ambient temperature. Solid state NMR spectra were recorded under magic angle spinning at 14.1 T using Bruker NEO600 spectrometer. All solid-state NMR samples were packed in 4 mm zirconia rotors and sealed with a Kel-F cap in an argon filled glovebox.

Matrix assisted laser desorption ionization (MALDI) mass spectrometry were recorded on an AB-SCIEX 5800 MALDI TOF/TOF mass spectrometer. Samples were prepared by dissolving extracted oil (5 mg) in THF (5 mL). Prior to spotting on the sample plate, an aliquot of this solution (0.1 mL) was mixed with a saturated solution of $AgNO_3$ in MeCN (0.1 mL). ~ 0.5 µL of the solution was placed on the sample plate, followed by 0.5 µL of the matrix solution (2,5 dihydroxybenzoic acid (DHB) solution prepared in a 3:2 (*v*:*v*) mixture of tetrahydrofuran and methanol at a 10 mg/mL concentration). The solvents were removed by gently heating the stainless-steel sample plate under air.

Synthesis of Cp_2HfMe_2/≡SiOAl(OC(CF_3)$_3$)$_2$(O(Si≡)$_2$): ≡SiOAl(OC(CF_3)$_3$)$_2$(O(Si≡)$_2$) (0.500 g, 0.11 $mmol_{Al}$ g^{-1}) and Cp_2HfMe_2 (1 eq, 0.11 mmol, 0.037 g) were transferred to one arm of a double-Schlenk flask inside an argon-filled glovebox. The flask was removed from the glovebox, connected to a high vacuum line, and evacuated for 5 min. Pentane (~8 mL) was condensed onto the solids under vacuum at 77 K. The mixture was warmed to room temperature and stirred gently for 40 minutes. The clear, colorless solution was then filtered away from the solids to the other side of the double-Schlenk. The arm of the double-Schlenk containing the functionalized silica was cooled to 77K, causing the pentane on the other side of the flask to condense onto the solids. The mixture was warmed to 25˚C, stirred for 5 min, and filtered back to the other side of the double Schlenk. This procedure was repeated two more times to wash the functionalized silica of unreacted Cp_2HfMe_2. The volatiles were distilled into a separate large volume Schlenk flask (2 L) fitted with a Teflon-tap cooled to 77K under vacuum. Warming the flask to room temperature places all solvent and any CH_4 formed in this reaction into the gas phase. Analysis of the gas phase by GC-FID shows 0.07 ± 0.001 $mmol_{CH4}$ g^{-1} released during the grafting. The double-Schleck flask was dried under diffusion pump vacuum for 45 minutes. Cp_2HfMe_2/≡SiOAl(OC(CF_3)$_3$)$_2$(O(Si≡)$_2$) is a white solid. This material was stored in an Ar glovebox freezer at -20˚C. An identical procedure was used to prepare ^{13}C labeled $Cp_2Hf(^{13}CH_3)_2$/≡SiOAl(OC(CF_3)$_3$)$_2$(O(Si≡)$_2$). The white solid was stored in an Ar glovebox freezer at -20˚C. Digestion of this material in 2% nitric acid for 12 hours at room temperature gives 0.21 $mmol_{Hf}$ g^{-1} by ICP-EOS analysis. FT-IR: $\nu_{C\text{-}H}$ = 3120 and 2924 (C-H from Cp_2HfMe) cm^{-1}. ^1H MAS NMR (10 kHz, -20 ºC): δ 5.7 ppm (Cp), 0.2 ppm (Hf-Me/Al-Me) ; $^{13}C\{^1H\}$ CPMAS NMR (10 kHz, -20 ºC): δ 112 (Cp), 38 (Hf-Me^+), 24 (Hf-Me), 2 Si-Me, -11 (Al-Me^-) ppm.

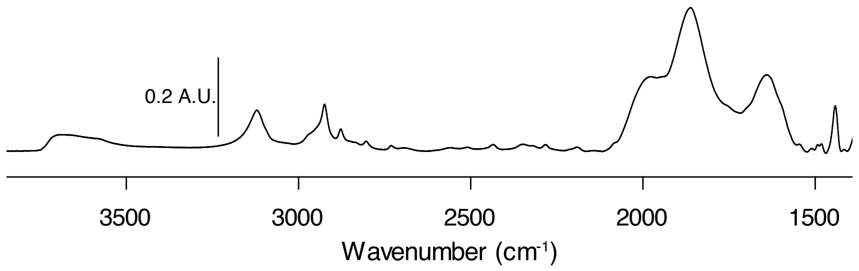

Figure S1. FT-IR spectrum of Cp_2HfMe_2/≡SiOAl(OC(CF_3)$_3$)$_2$(O(Si≡)$_2$).

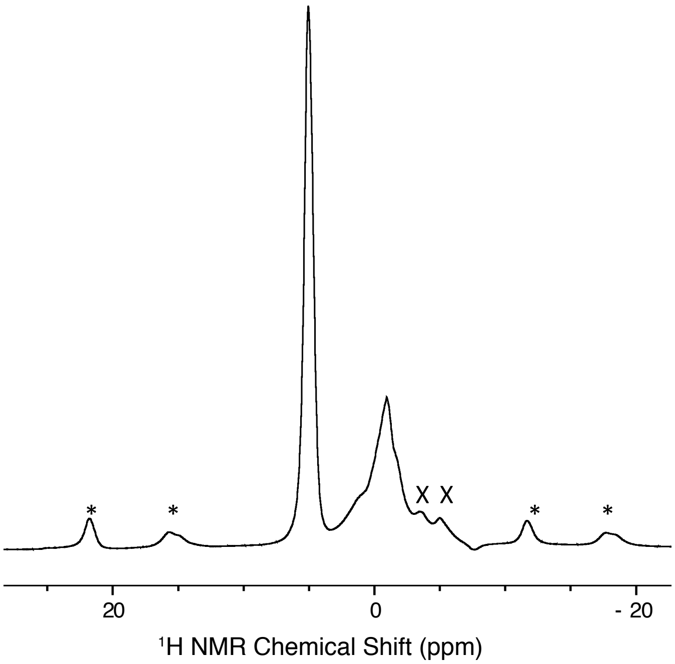

Figure S2. 10kHz MAS ^{1}H NMR of Cp_2HfMe_2/≡SiOAl(OC(CF_3)$_3$)$_2$(O(Si≡)$_2$) acquired at -20˚C. Probe background signals are labeled with an "x," spinning sidebands are labeled with a *.

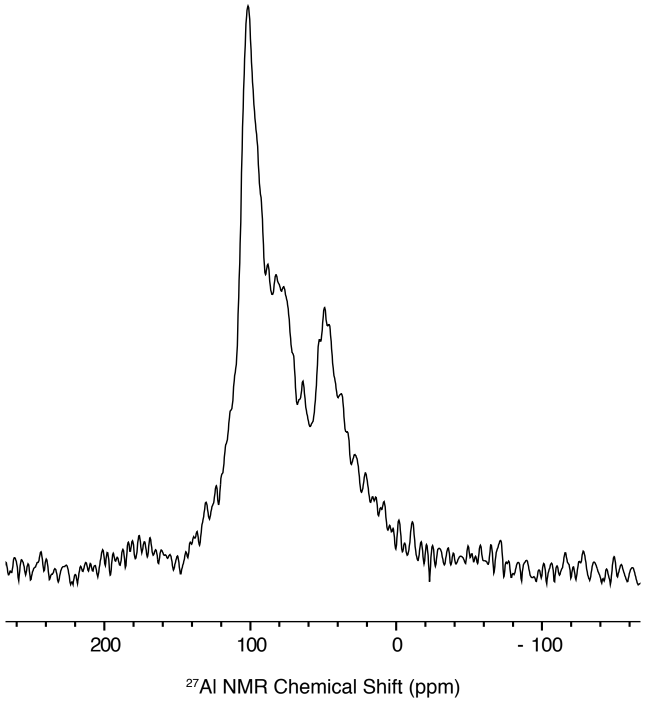

Figure S3. ^{27}Al{^{1}H} MAS NMR spectrum of Cp_2HfMe_2/≡SiOAl(OC(CF_3)$_3$)$_2$(O(Si≡)$_2$) recorded at 10 kHz spinning speed.

Synthesis of $Cp_2Hf(^{13}CH_3)(OSi\equiv)$ (2): Silica partially dehydroxylated at 700 °C (SiO_{2-700}, 0.500 g, 0.13 mmol -OH) and Cp_2HfMe_2 (1 eq, 0.13 mmol, 0.044 g) were transferred to one arm of a double-Schlenk flask inside an argon-filled glovebox. The flask was removed from the glovebox, connected to a high vacuum line, and evacuated for 5 min. Pentane (~ 8 mL) was condensed onto the solids under vacuum at 77 K. The mixture was warmed to room temperature and stirred gently for 40 minutes. The clear, colorless solution was then filtered away from the solids to the other side of the double-Schlenk. The arm of the double Schlenk containing the functionalized silica was cooled to 77K, causing the pentane on the other side of the flask to condense onto the solids. The mixture was warmed to 25˚C, stirred for 5 min, and filtered back to the other side of the double Schlenk. This procedure was repeated two more times to wash the functionalized silica of unreacted Cp_2HfMe_2. The volatiles were distilled into a separate large volume Schlenk flask (2 L) fitted with a Teflon-tap cooled to 77K under vacuum. Warming the flask to room temperature places all solvent and any CH_4 formed in this reaction into the gas phase. Analysis of the gas phase by GC-FID shows 0.25 $mmol_{CH4}$ g^{-1} released during the grafting. The double-Schleck flask was dried under diffusion pump vacuum for 45 minutes. The white solid was stored in an Ar glovebox freezer at -20˚C. FT-IR: $\nu_{C\text{-}H}$ = 3115 and 2915 (C-H from Cp_2HfMe) cm^{-1}. ^1H MAS NMR (10 kHz, -20 °C): δ 5.5 (CpH), 0.18 (Hf–CH_3); $^{13}C\{^1H\}$ CPMAS NMR (10 kHz, -20 °C): δ 110 (Cp), 23 (Hf-Me) ppm.

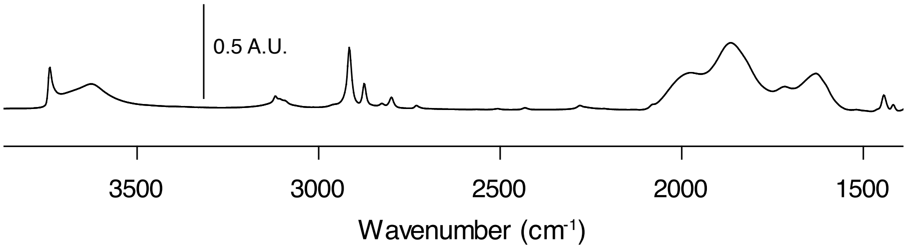

Figure S4. FT-IR spectrum of **2** wavenumbers (cm^{-1}).

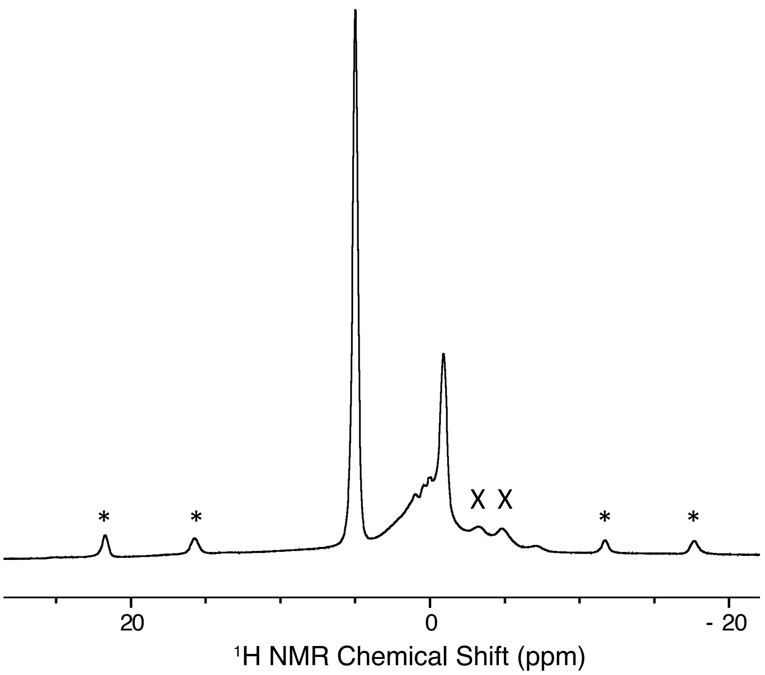

Figure S5. ^{1}H NMR of **2** acquired at -20˚C and 10 kHz spinning speed. Probe background signals are labeled with an "x," spinning sidebands are labeled with a *.

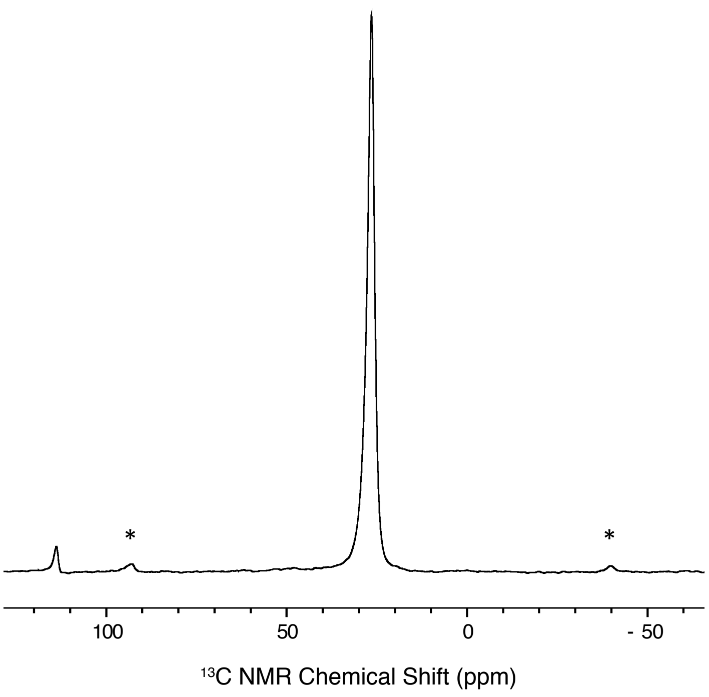

Figure S6. $^{13}C\{^1H\}$ CPMAS NMR of **2** acquired at -20˚C at 10 kHz spinning speed. Spinning sidebands are labeled with a *.

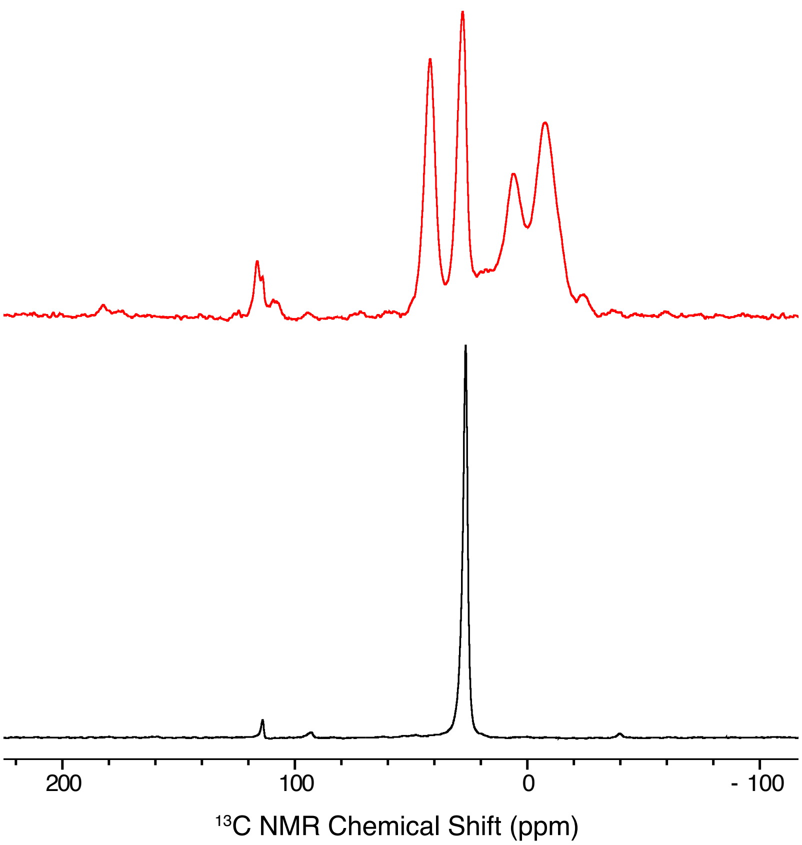

Figure S7. Stacked plot of the $^{13}C\{^1H\}$ CPMAS NMR spectrum of Cp_2HfMe_2/≡SiOAl(OC(CF_3)$_3$)$_2$(O(Si≡)$_2$) (top, red) and **2** (bottom, black).

Reaction of Cp_2HfMe_2/≡SiOAl(OC(CF_3)$_3$)$_2$(O(Si≡)$_2$) or $Cp_2Hf(CH_3)$(OSi≡) with H_2.

In an argon-filled glovebox, a 100mL Schlenk flask fitted with a Teflon-tap was loaded with 200 mg $Cp_2Hf(CH_3)_2$/≡SiOAl(OC(CF_3)$_3$)$_2$(O(Si≡)$_2$) (0.042 mmol Hf). The flask was removed from the glovebox, connected to a high vacuum line, and evacuated for 5 min. The flask was filled with 1atm of H_2 (4.16 mmol), sealed, disconnected from the line, and heated at 150 ˚C for 12h. Volatiles were sampled directly from the flask and analyzed by GC FID (0.09 mmol CH_4/g). The flask was evacuated and the material was stored in an argon-filled glovebox freezer. An identical procedure was used for reaction of $Cp_2Hf(CH_3)$(OSi≡) (0.052 mmol Hf) with 1atm of H_2 (4.16 mmol). The flask was filled with 1atm of H_2 (4.16 mmol), sealed, disconnected from the line, and heated at 150 ˚C for 12h. Volatiles were sampled directly from the flask and analyzed by GC FID (0.001 mmol CH_4/g).

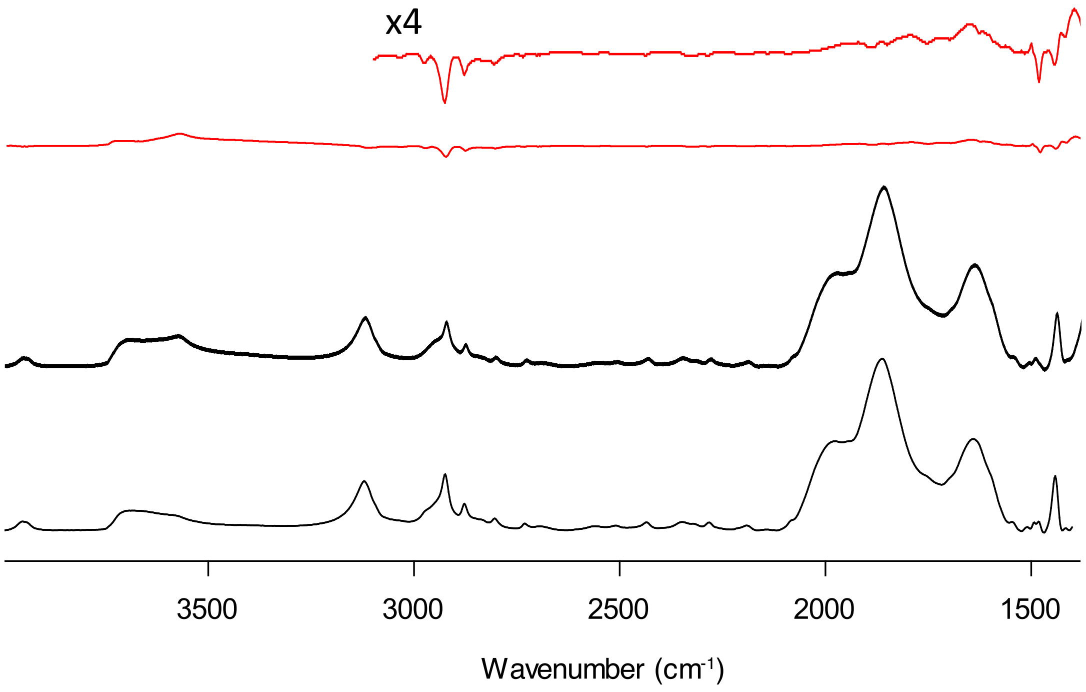

Figure S8. FTIR of Cp_2HfMe_2/≡SiOAl(OC(CF_3)$_3$)$_2$(O(Si≡)$_2$) treated with H_2 at 150 °C (middle, black), FTIR of Cp_2HfMe_2/≡SiOAl(OC(CF_3)$_3$)$_2$(O(Si≡)$_2$) (bottom), and the difference spectrum showing which FTIR signals disappear and appear in this reaction (top, red trace and close up).

Procedure for the hydrogenolysis of iPP with Cp_2HfMe_2/≡SiOAl($OC(CF_3)_3)_2$(O(Si≡$)_2$).

At 1 atm:

In an argon-filled glovebox, a 100mL Schlenk flask fitted with a Teflon-tap was loaded with 200 mg iPP and 200 mg $Cp_2Hf(CH_3)_2$/≡SiOAl($OC(CF_3)_3)_2$(O(Si≡$)_2$) (0.042 mmol Hf). The flask was removed from the glovebox, connected to a high vacuum line, and evacuated for 5 min. The flask was filled with 1atm of H_2 (4.16 mmol), sealed, disconnected from the line, and heated at 200 ˚C for 24h. Volatiles were sampled directly from the flask and analyzed by GC FID. Following analysis of volatile gases, the flask was opened to ambient atmosphere to proceed with the extraction of oils and remaining solids. Dichloromethane (~10 mL) was added to the flask at room temperature, and the solution was decanted from the residual polymer melt and spent catalyst mixture. This was repeated two more times. The combined dichloromethane extract was concentrated by heating gently to remove the solvent.

An identical procedure was used for reactions of iPP with D_2, except purified D_2 was used in place of H_2. An essentially identical procedure was used in experiments with H_2:Hf(Zr) ~ 1500, but a 1.5L glass bottle as shown below was used. The polymer and the catalyst were placed in the nub on the bottom of the flask and heated under 1 atm H2.

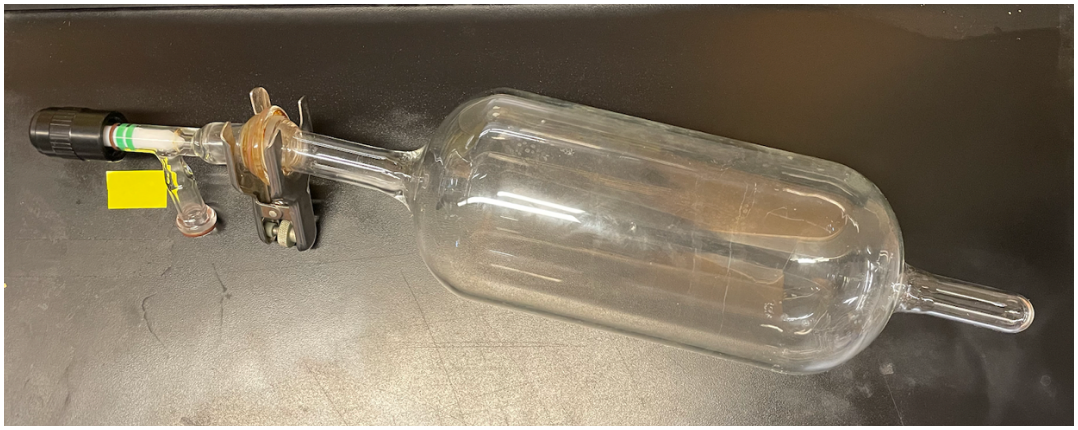

At 5 or 10atm Pressure in a Parr Reactor:

A 100 mL glass reaction liner was charged with 200 mg of $Cp_2Hf(CH_3)_2$/≡SiOAl($OC(CF_3)_3)_2$(O(Si≡$)_2$) and 200 mg iPP in an argon filled glovebox. The reactor was sealed and pressurized with desired pressure of hydrogen (passed through a CRS ZPure O_2/H_2O filter) on demand and heated at 300 °C for 24h. The higher temperature is necessary because the thermocouple is not measuring temperature in the glass sleave. Control experiments showed that 300 °C is required to melt iPP; 200 °C to 250 °C was not hot enough to melt the polymer. After the reaction, the reactor was cooled to ambient temperature and the volatile gases were transferred into a 2L flask. Gas samples were aliquoted to determine volatile gas yields. CH_2Cl_2 (10 mL) was added to each glass liner under ambient atmosphere, and the solution was decanted from the remaining solid. This procedure was repeated three more times. The combined CH_2Cl_2 extract was

concentrated by heating gently to remove the solvent and yields were calculated by weighing the amount of oil isolated.

At 2 or 5atm Pressure fed on Demand in a Parallel High Pressure Reactor:

Hydrogenolysis of iPP reactions at elevated H_2 pressures on demand were performed in a Biotage Endeavor parallel reactor in a N_2 filled glovebox. A 15 mL glass reaction liner was charged with 200 mg of $Cp_2Hf(CH_3)_2$/≡SiOAl(OC(CF_3)$_3$)$_2$(O(Si≡)$_2$) and 200 mg iPP. The reactor was sealed and pressurized with desired pressure of hydrogen on demand and heated at 200 °C for 24h. After the reaction the reactor was vented with N_2 and cooled to ambient temperature inside the glovebox. CH_2Cl_2 (10 mL) was added to each glass liner under ambient atmosphere, and the solution was decanted from the remaining solid. This procedure was repeated three more times. The combined CH_2Cl_2 extract was concentrated by heating gently to remove the solvent and yields were calculated by weighing the amount of oil isolated.

Quantification of Gas Phase Products from the hydrogenolysis of iPP with Cp_2HfMe_2/≡SiOAl($OC(CF_3)_3)_2$(O(Si≡)$_2$)

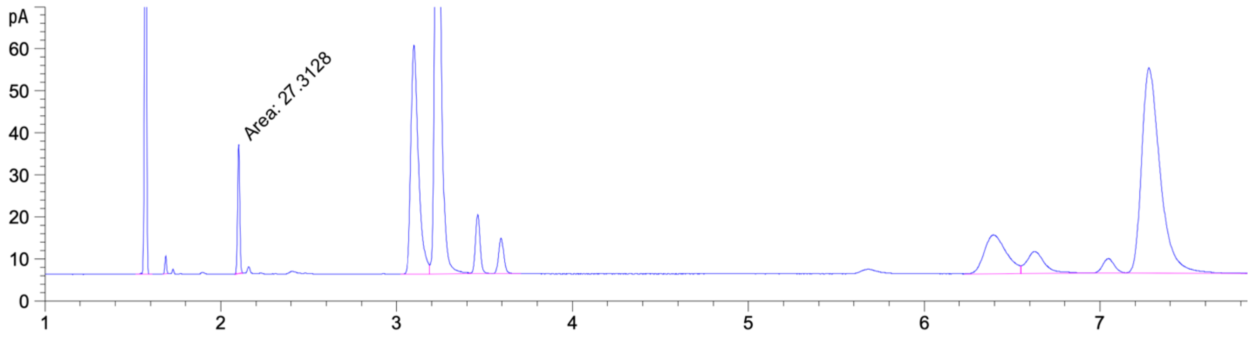

Figure S9. GC of the gas phase of iPP hydrogenolysis reactions with Cp_2HfMe_2/≡SiOAl($OC(CF_3)_3)_2$(O(Si≡)$_2$) under 1 atm H_2 (H_2:Hf ~ 100). The amounts gases are reported in the main text.

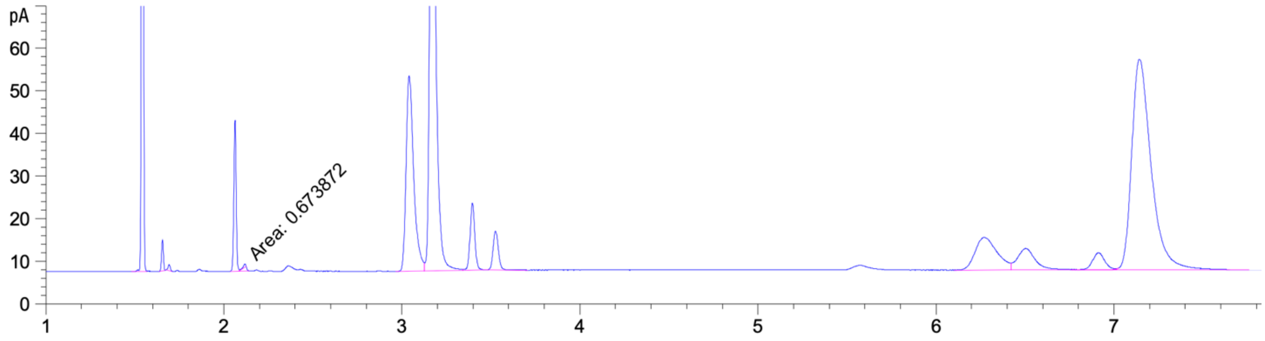

Figure S10. GC of the gas phase of iPP hydrogenolysis reactions with Cp_2HfMe_2/≡SiOAl($OC(CF_3)_3)_2$(O(Si≡)$_2$) under 1 atm D_2 (D_2:Hf ~ 100). The amounts gases generated in this reaction are essentially identical to those reported in the main text.

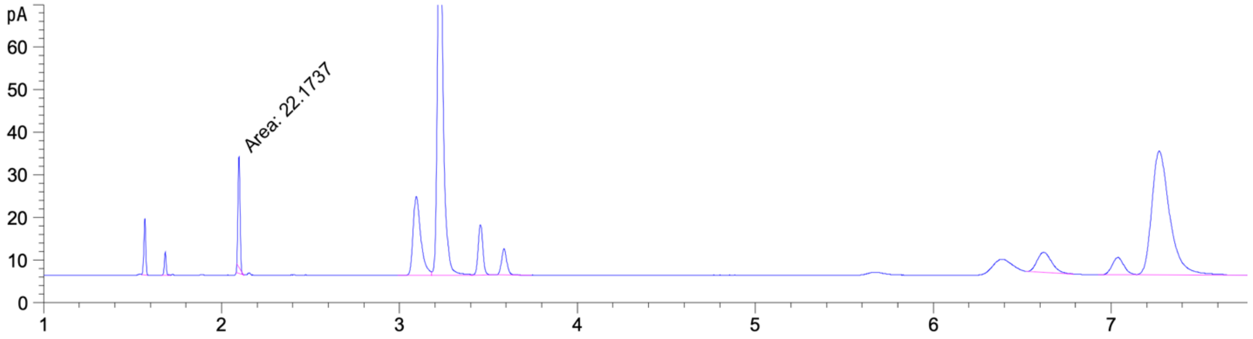

Figure S11. GC of the gas phase of iPP hydrogenolysis reactions with Cp_2HfMe_2/≡SiOAl($OC(CF_3)_3)_2$(O(Si≡)$_2$) under 1 atm H_2 (H_2:Hf ~ 1500). The amounts of gas evolved are 1.8 CH_4 Hf^{-1}, 0.4 C_2H_6 Hf^{-1}, 1.35 C_3H_6 Hf^{-1} and 9.9 C_4H_{10} Hf^{-1}, and 9.3 C_5H_{10} Hf^{-1}. The total yield of light gases are 11.5 % (23 mg) based on this data.

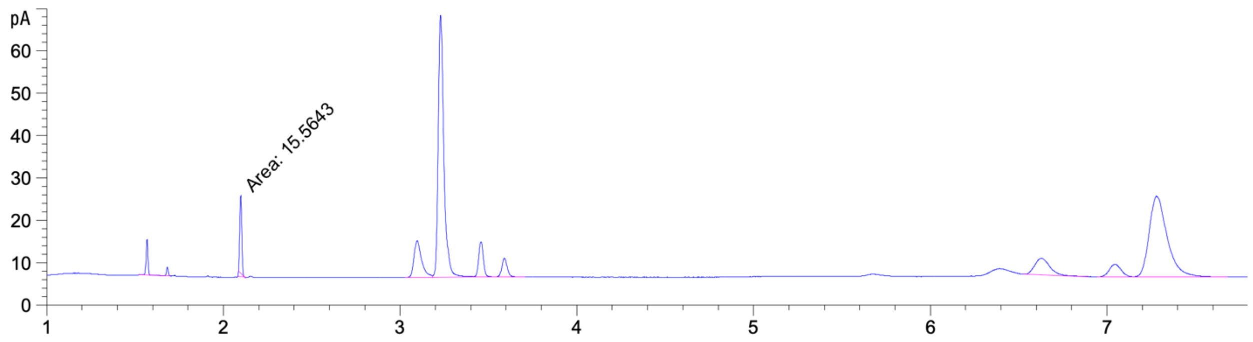

Figure S12. GC of the gas phase of iPP hydrogenolysis reactions with Cp_2ZrMe_2/≡SiOAl(OC(CF_3)$_3$)$_2$(O(Si≡)$_2$) under 1 atm H_2 (H_2:Zr ~ 1500). The amounts of gas evolved are 1.1 CH_4 Hf^{-1}, 1.6 C_2H_6 Zr^{-1}, 1.0 C_3H_6 Zr^{-1} and 6.7 C_4H_{10} Zr^{-1}, and 4.9 C_5H_{10} Zr^{-1}. The total yield of light gases are 7 % (14 mg) based on this data.

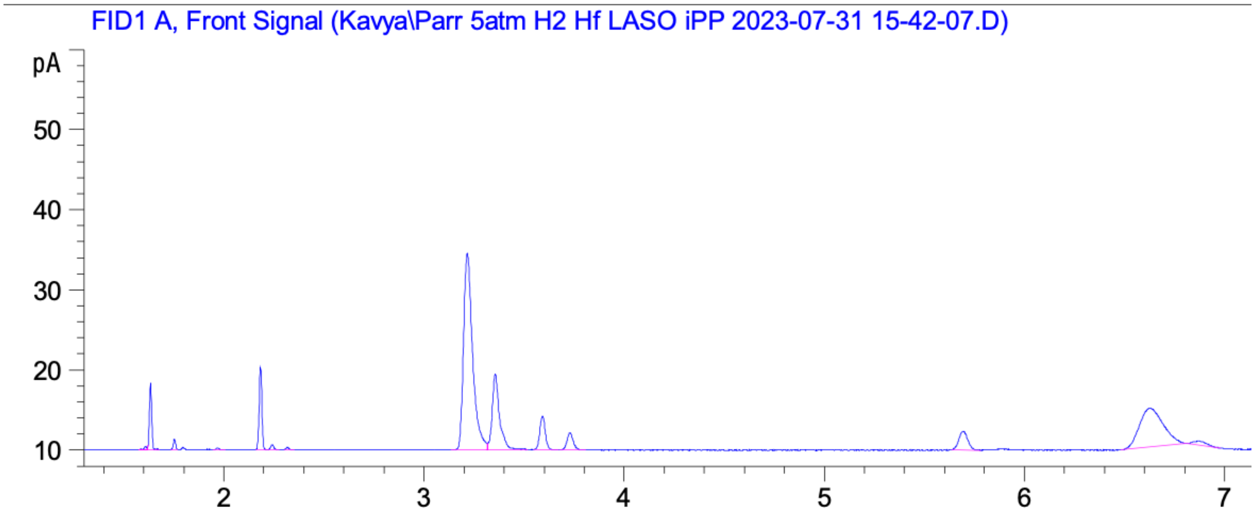

Figure S13. GC of the gas phase of iPP hydrogenolysis reactions with Cp_2HfMe_2/≡SiOAl(OC(CF_3)$_3$)$_2$(O(Si≡)$_2$) under 5 atm H_2. The amounts of gas evolved are 1.55 CH_4 Hf^{-1}, 0.12 C_2H_6 Hf^{-1}, 0.76 C_3H_6 Hf^{-1}, 6.62 C_4H_{10} Hf^{-1}, and 1.81 C_5H_{10} Hf^{-1}. The total yield of light gases are 4 % (8 mg) based on this data.

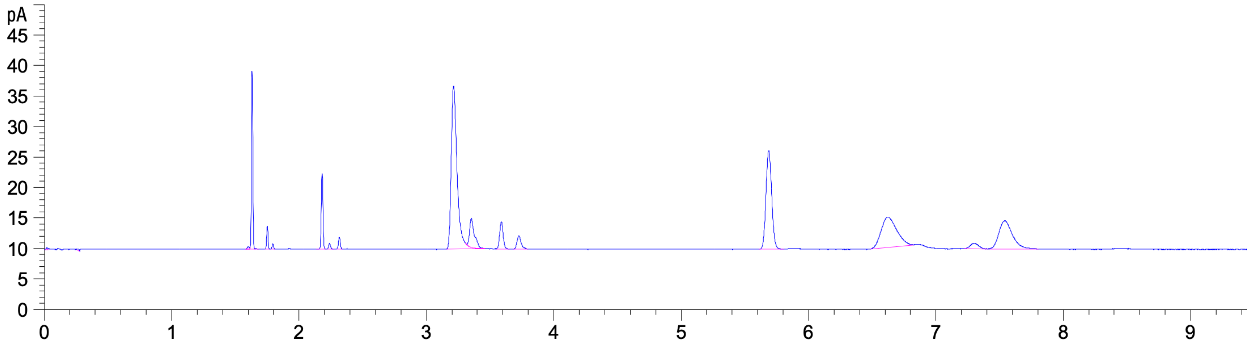

Figure S14. GC of the gas phase of iPP hydrogenolysis reactions with $Cp_2HfMe_2/\equiv SiOAl(OC(CF_3)_3)_2(O(Si\equiv)_2)$ under 10 atm H_2. The amounts of gas evolved are 5.4 CH_4 Hf^{-1}, 0.37 C_2H_6 Hf^{-1}, 1.1 C_3H_6 Hf^{-1}, 6.83 C_4H_{10} Hf^{-1}, and 6.53 C_5H_{10} Hf^{-1}. The total yield of light gases are 7.3 % (14.6 mg) based on this data.

MALDI MS of oils from the hydrogenolysis of iPP with Cp_2HfMe_2/≡SiOAl(OC(CF_3)$_3$)$_2$(O(Si≡)$_2$).

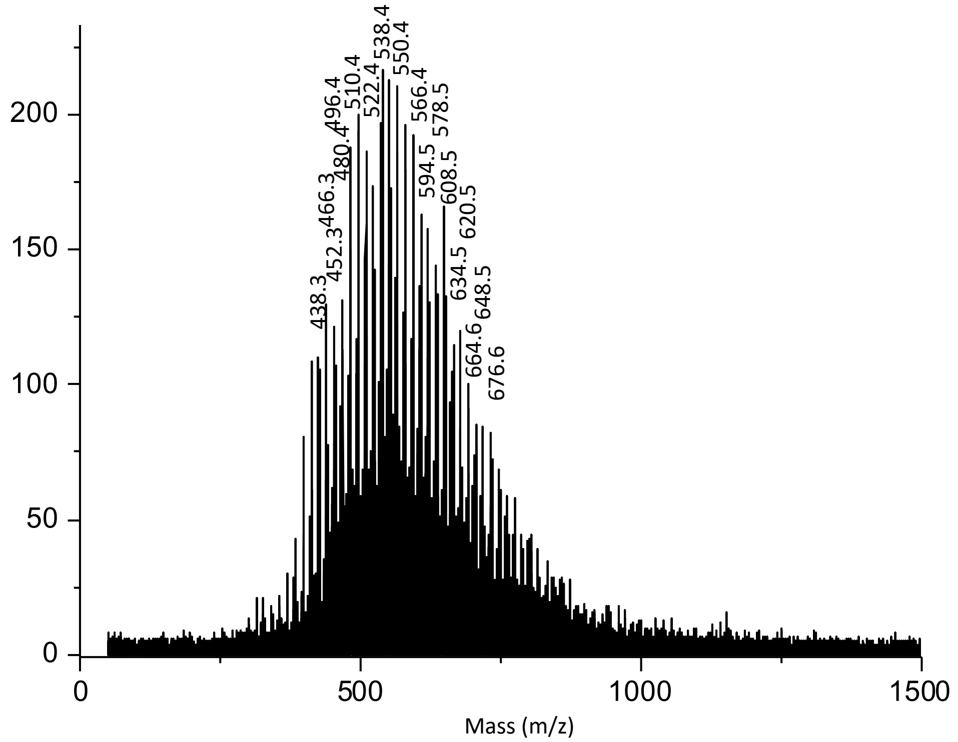

Figure S15. Representative MALDI MS of the oils obtained from hydrogenolysis of iPP with H_2:Hf ~ 100.

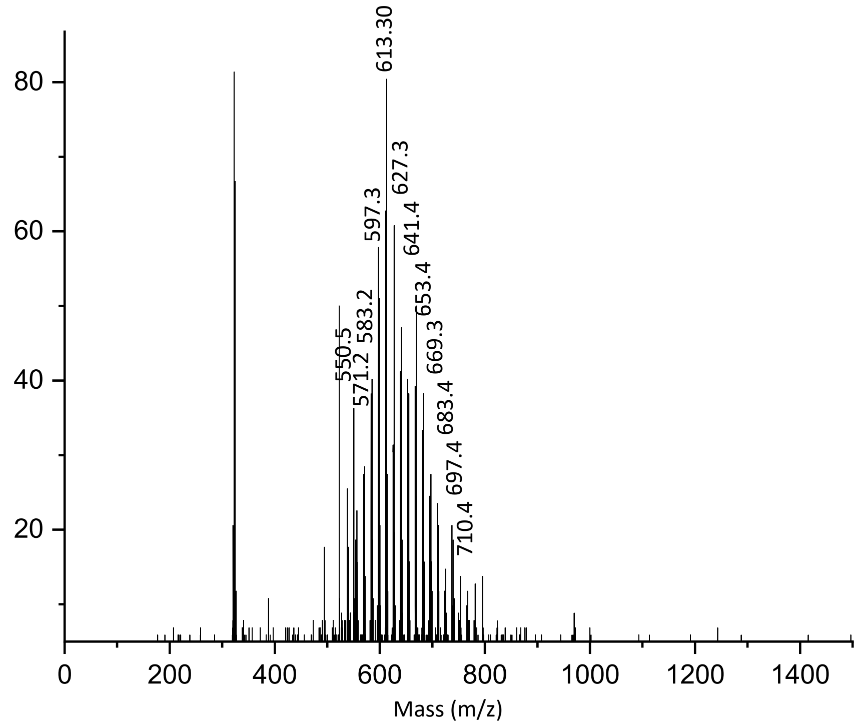

Figure S16. Representative MALDI MS of the oils obtained from hydrogenolysis of iPP with D_2:Hf ~ 100.

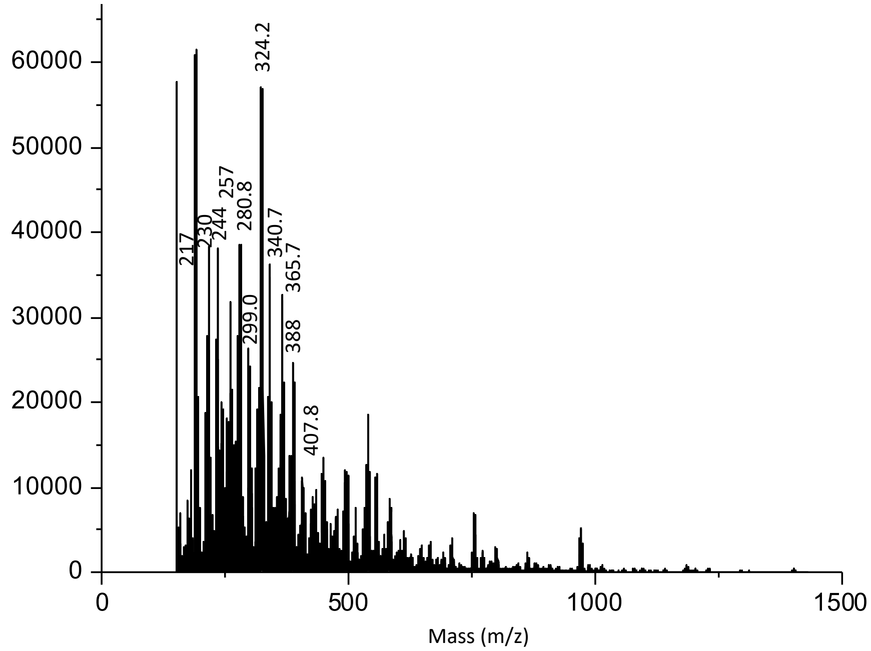

Figure S17. Representative MALDI MS of the oils obtained from hydrogenolysis of iPP with H_2:Hf ~ 1500.

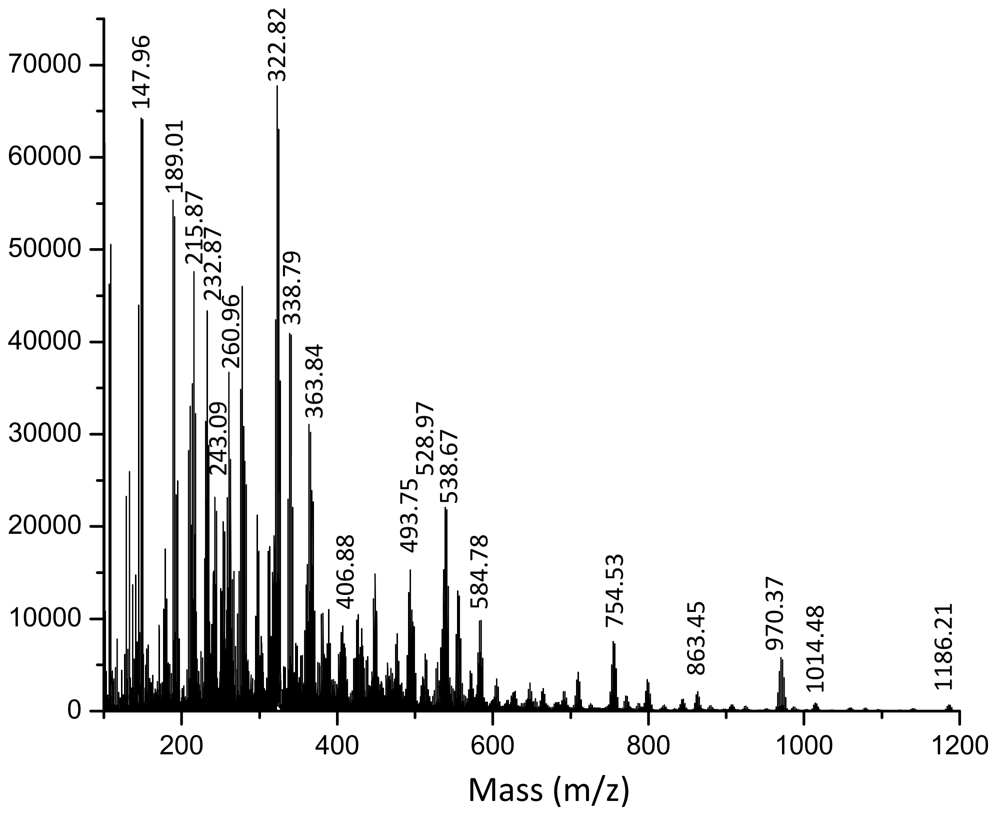

Figure S18. Representative MALDI MS of the oils obtained from hydrogenolysis of iPP under 5 atm H_2.

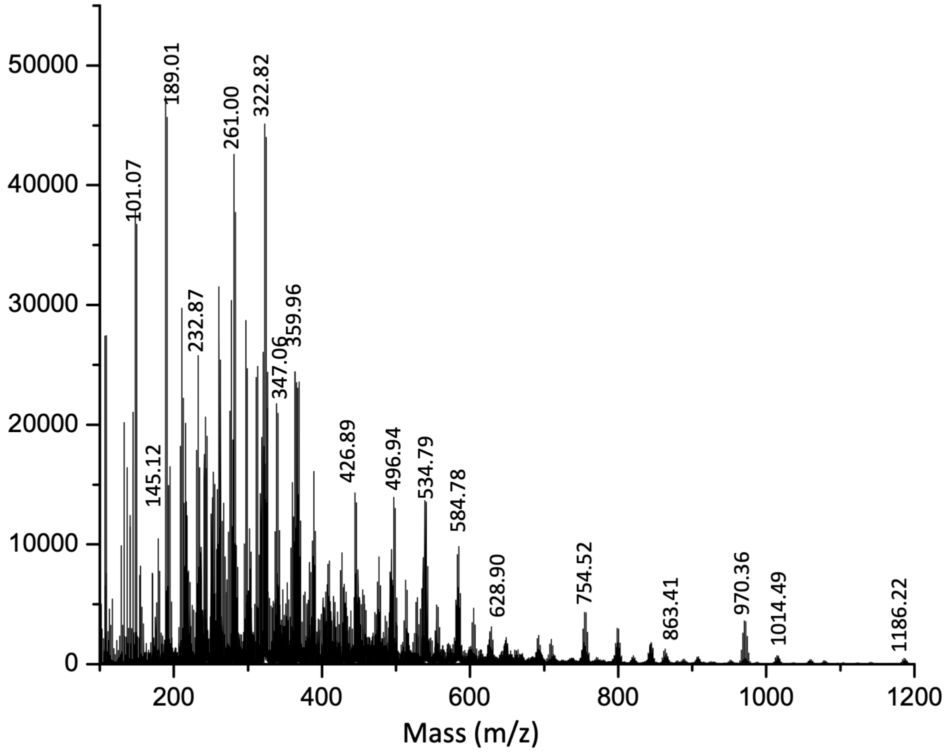

Figure S19. Representative MALDI MS of the oils obtained from hydrogenolysis of iPP under 10 atm H_2.

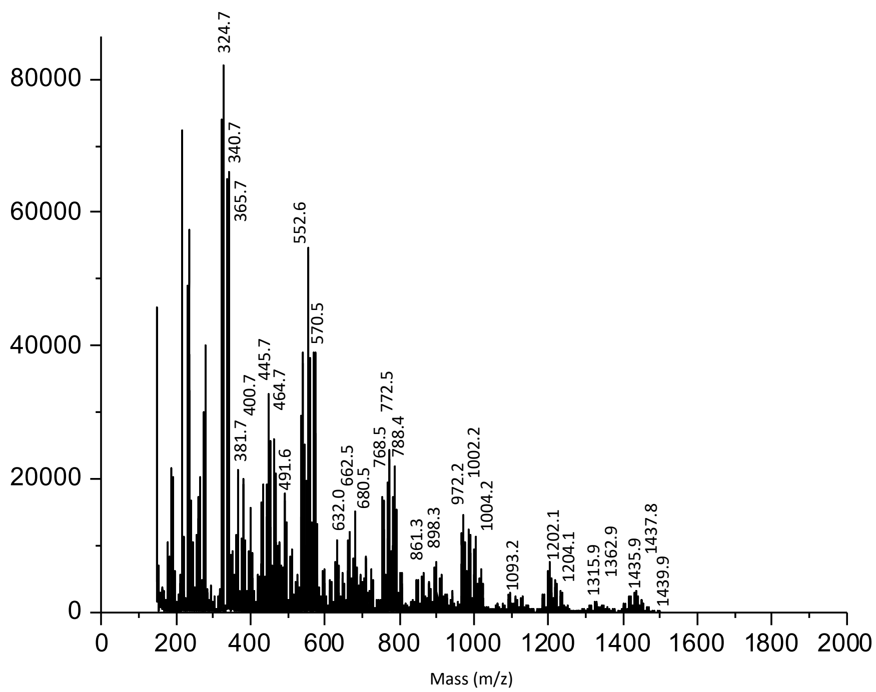

Figure S20. Representative MALDI MS of the oils obtained from hydrogenolysis of iPP under 2 atm H_2 fed on demand.

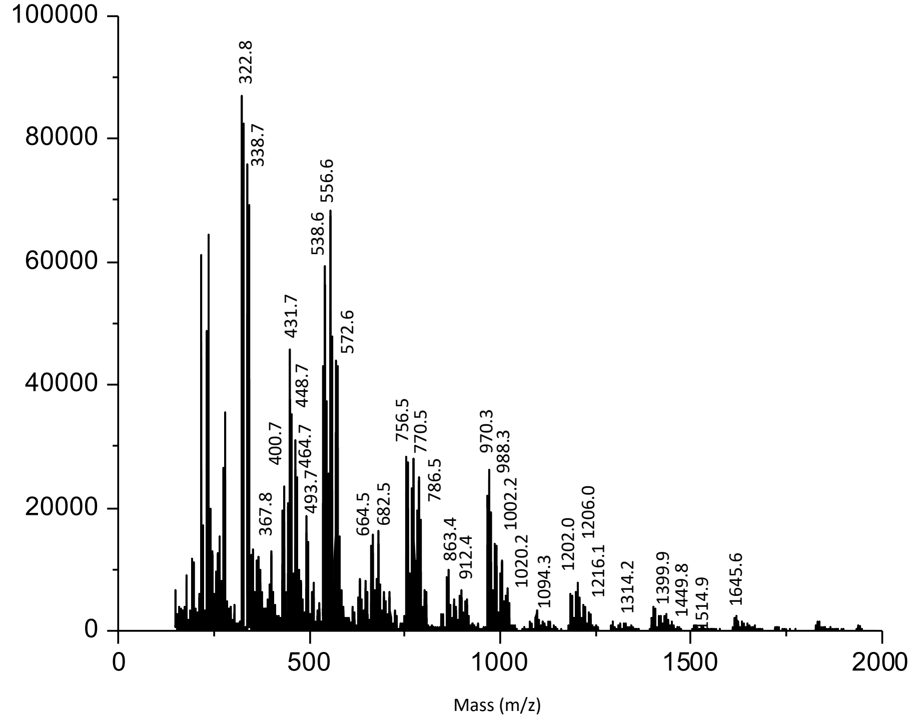

Figure S21. Representative MALDI MS of the oils obtained from hydrogenolysis of iPP under 5 atm H_2 fed on demand.

GCMS of oils from the hydrogenolysis of iPP with $Cp_2HfMe_2/{\equiv}SiOAl(OC(CF_3)_3)_2(O(Si{\equiv})_2)$.

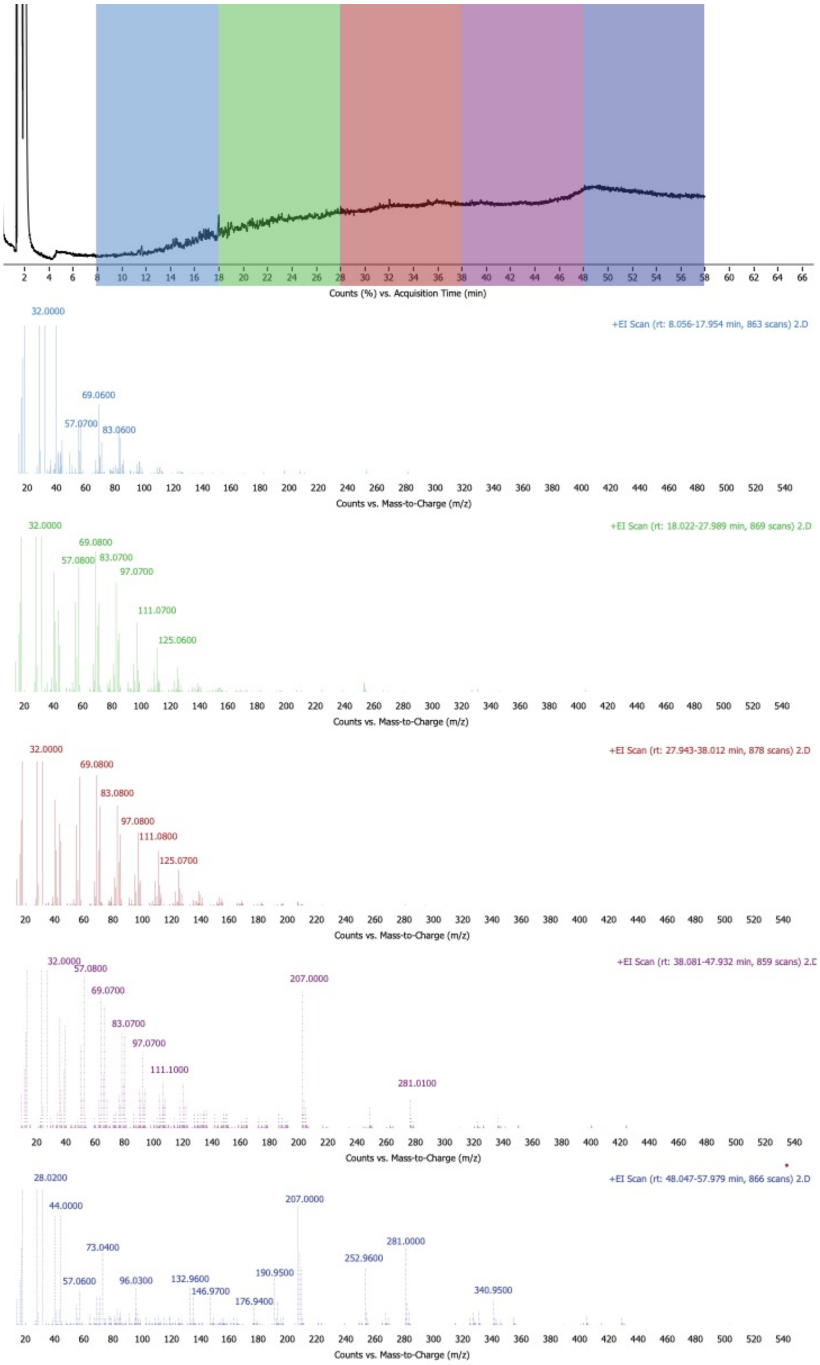

Figure S22. GCMS of oil produced with $Cp_2HfMe_2/{\equiv}SiOAl(OC(CF_3)_3)_2(O(Si{\equiv})_2)$ under 1 atm H_2 (H_2:Hf ~ 100). The data is too complex to integrate a single peak. The GC and MS data is color coded to give representative fractions showing how higher MW ions form at higher retention times.

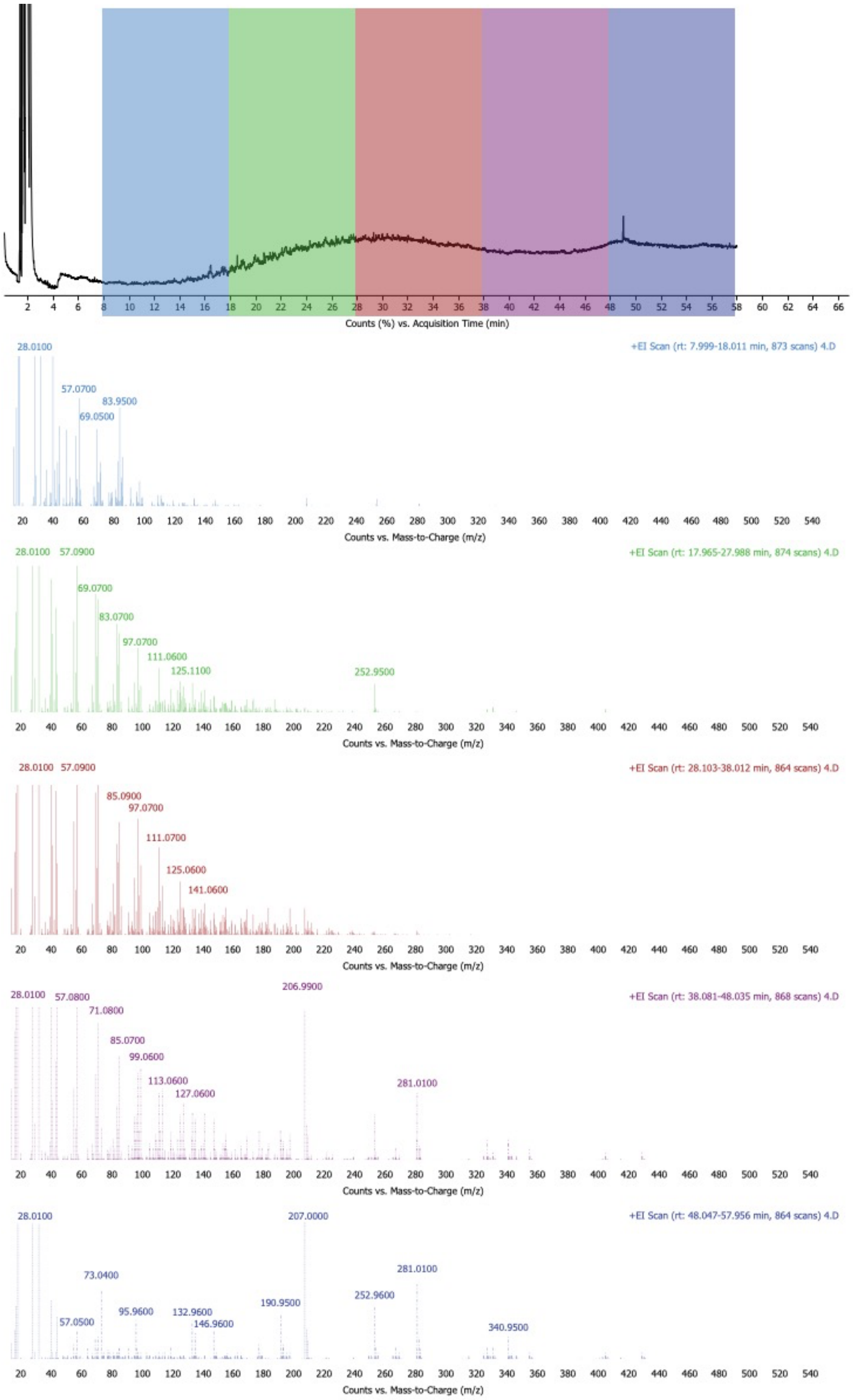

Figure S23. GCMS of oil produced with $Cp_2HfMe_2/{\equiv}SiOAl(OC(CF_3)_3)_2(O(Si{\equiv})_2)$ under 5 atm H_2. The data is too complex to integrate a single peak. The GC and MS data is color coded to give representative fractions showing how higher MW ions form at higher retention times.

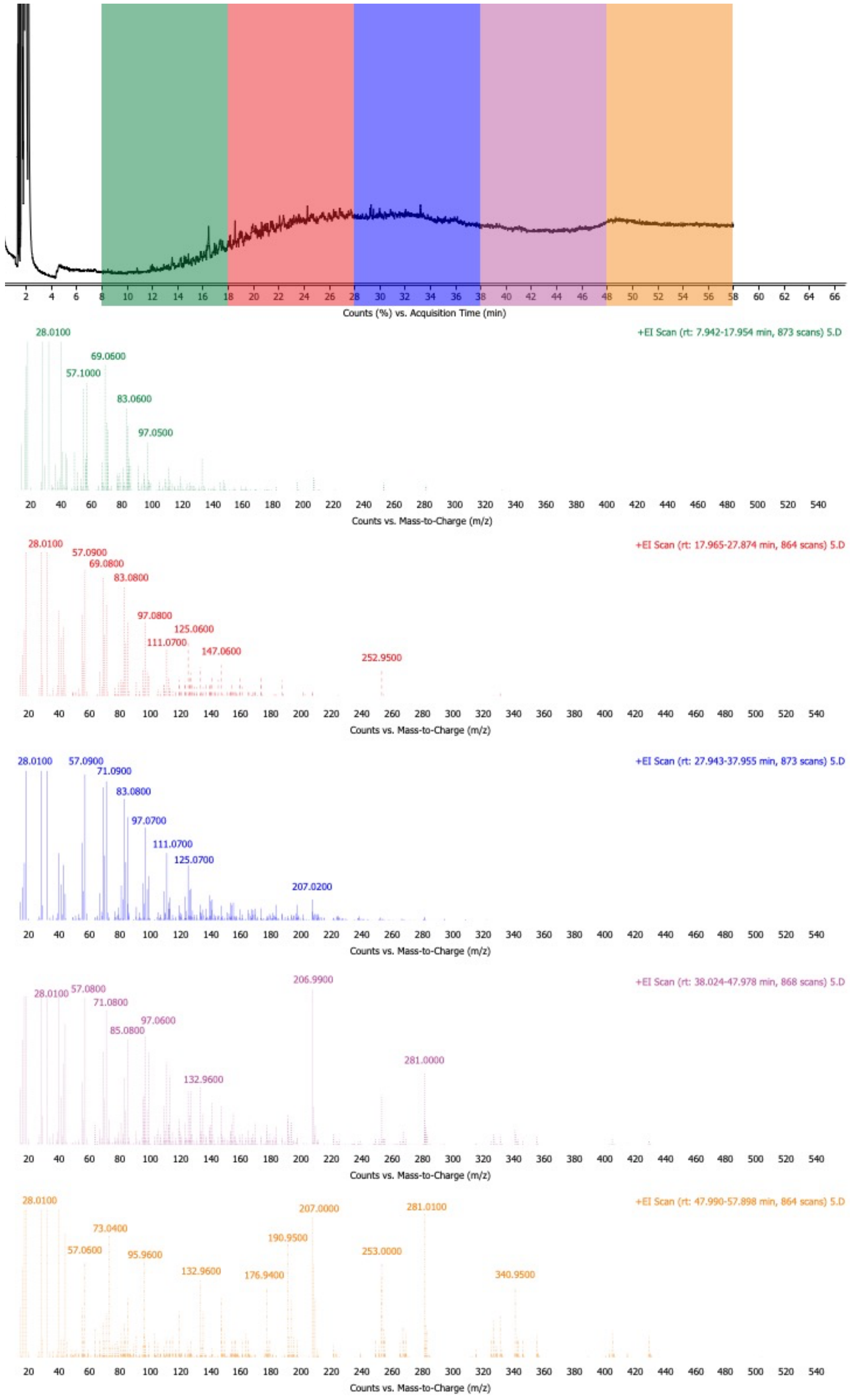

Figure S24. GCMS of oil produced with Cp_2HfMe_2/≡SiOAl(OC(CF_3)$_3$)$_2$(O(Si≡)$_2$) under 10 atm H_2. The data is too complex to integrate a single peak. The GC and MS data is color coded to give representative fractions showing how higher MW ions form at higher retention times.

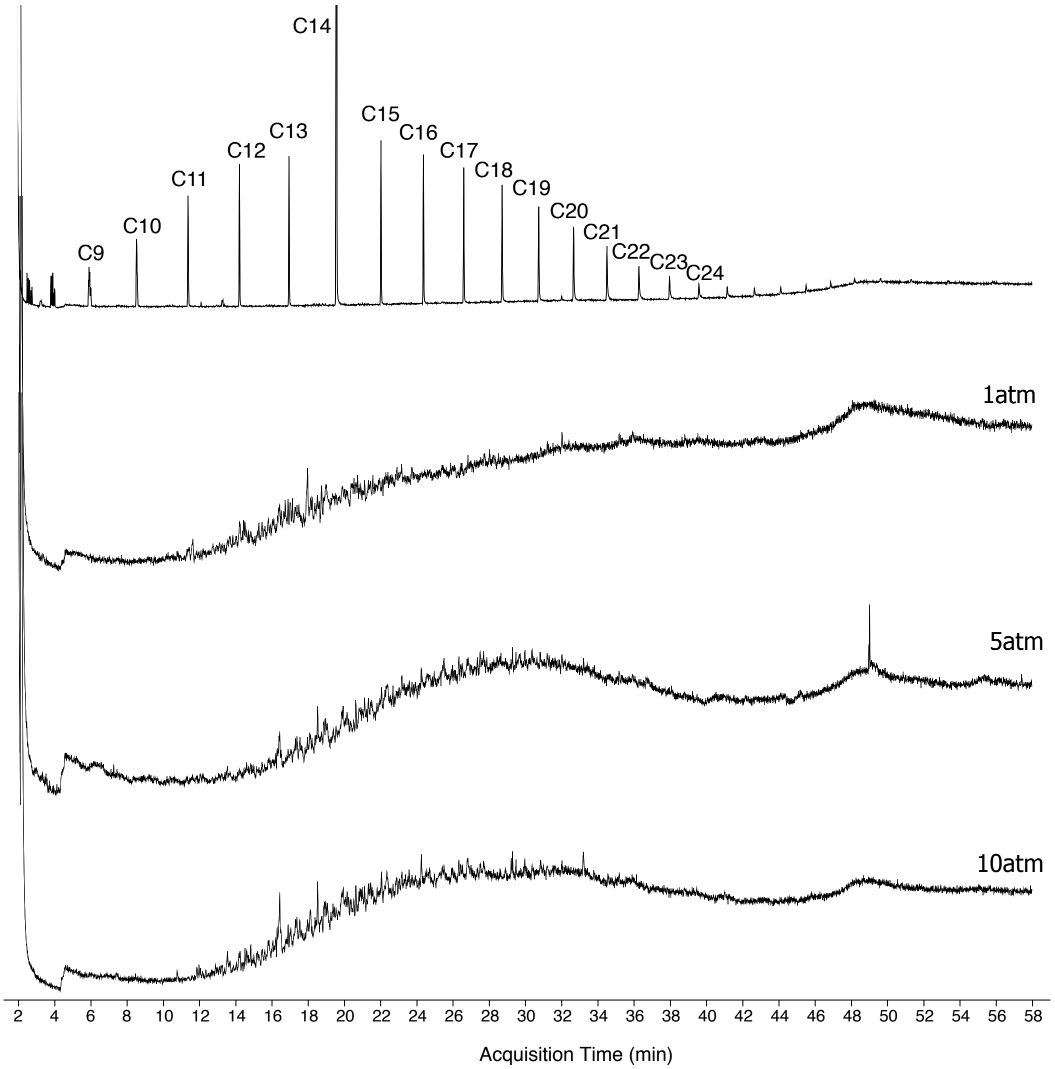

Figure S25. GC data of oils produced with Cp_2HfMe_2/≡SiOAl(OC(CF_3)$_3$)$_2$(O(Si≡)$_2$) under the pressures given in the figure (bottom three chromatograms). The top chromatogram is a mixture of alkanes produced from alkane metathesis reactions of tetradecane with a different catalyst (see: Gao, J.; Zhu, L.; Conley, M. P. Cationic Tantalum Hydrides Catalyze Hydrogenolysis and Alkane Metathesis Reactions of Paraffins and Polyethylene. *J. Am. Chem. Soc.* **2023**, *145*, 4964-4968). The top chromatogram contains linear the C_n listed in the figure to show the reader where linear alkanes appear with this method. Also apparent in this top chromatogram is the flat base line throughout the GC method, indicating the complexity of the oils produced in iPP hydrogenolysis reactions using this catalyst.

^{1}H NMR data for oils produced from the hydrogenolysis of iPP with Cp_2HfMe_2/≡ SiOAl($OC(CF_3)_3)_2$(O(Si≡$)_2$).

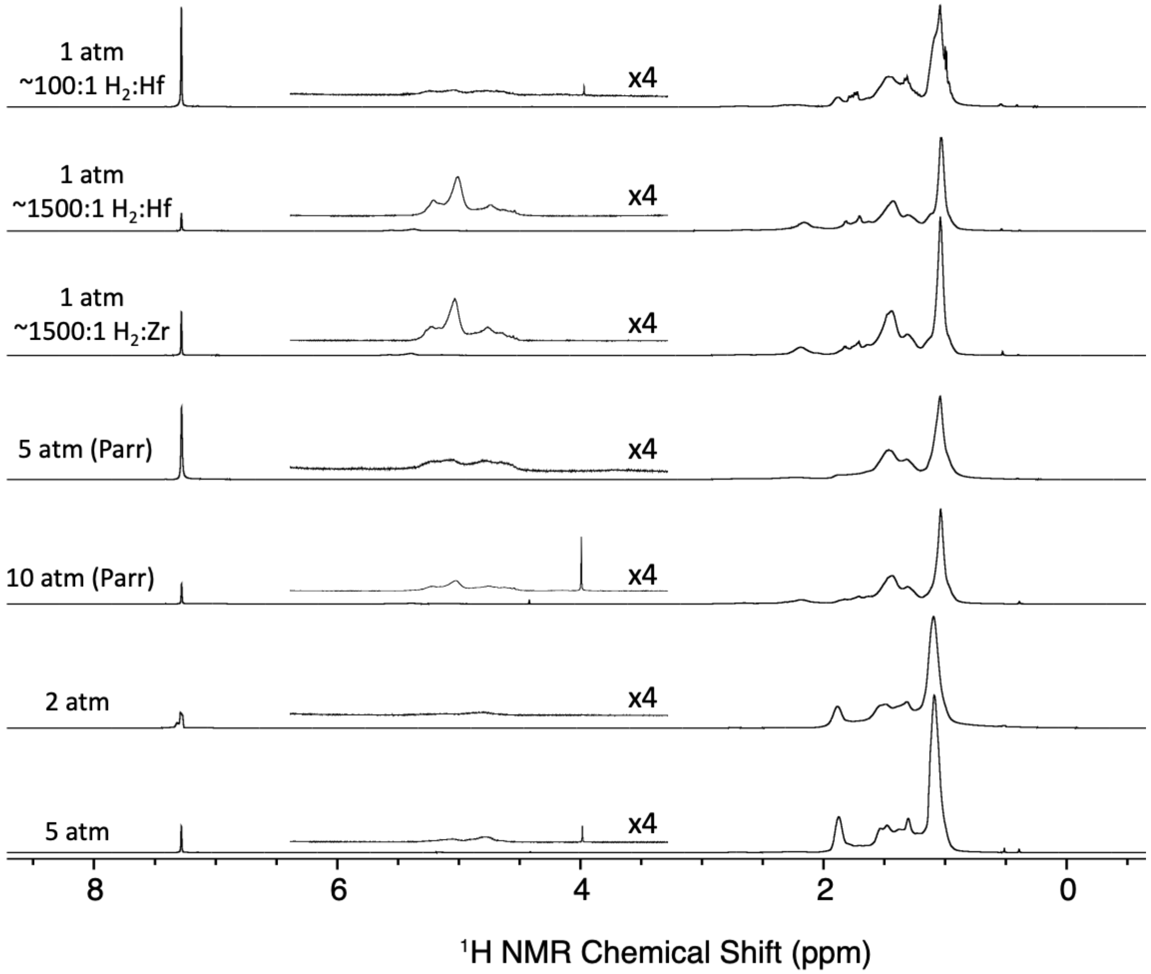

Figure S26. ^{1}H NMR spectra of extracted oils recorded in C_6D_6 at ambient temperature. The pressures in the figure correspond to the H_2 pressure used for the hydrogenolysis reaction.

Table S1. Integral values obtained from ^{1}H NMR data shown in Figure S17.

Conditions	Olefin:C_3H_6
1 atm (~100:1 H_2:Hf)	1:294
1 atm (~1500:1 H_2:Hf)	1:54
1 atm (~1500:1 H_2:Zr)	1:58
5 atm Parr	1:200
10 atm Parr	1:82
2 atm	1:1768
5 atm	1:533

^2H NMR data for using D_2 in iPP degradation reactions Cp_2HfMe_2/≡SiOAl(OC(CF$_3$)$_3$)$_2$(O(Si≡)$_2$).

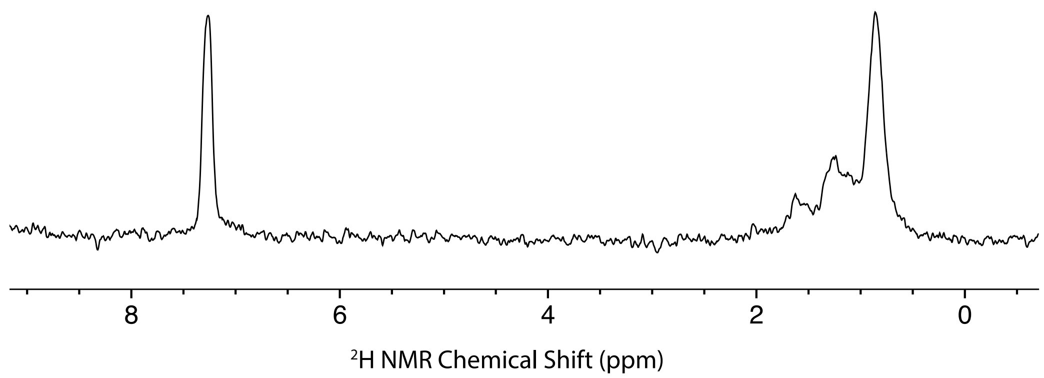

Figure S27. ^2H NMR of extracted oil recorded in $CHCl_3$ at ambient temperature. The oil in this spectrum was generated at D_2:Hf ~ 100.

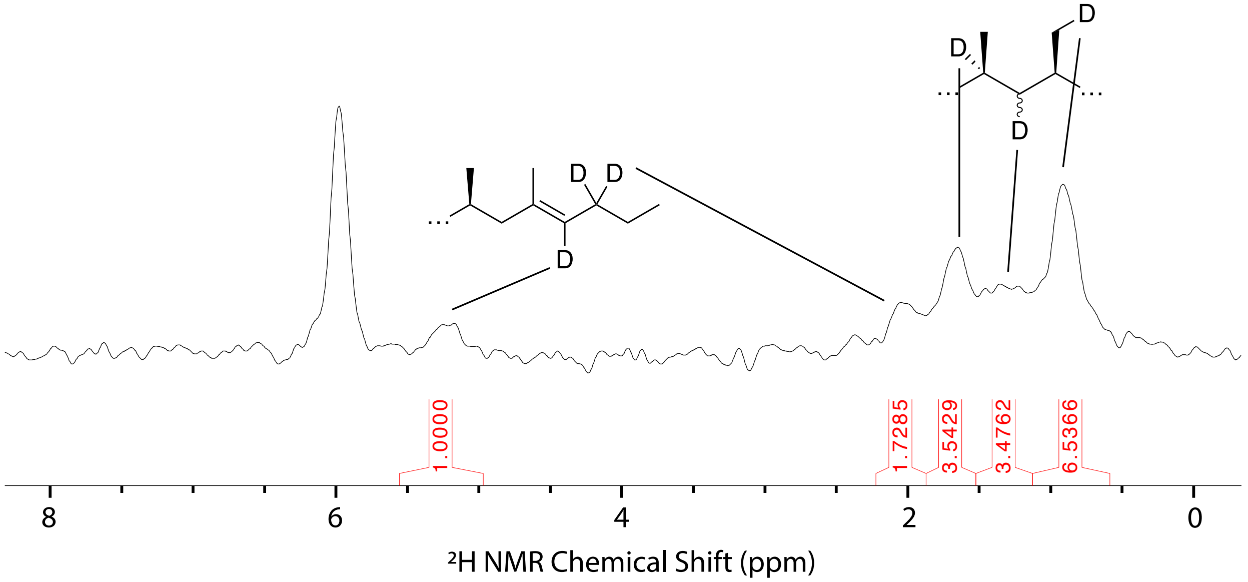

Figure S28. ^2H NMR spectrum of residual iPP after reaction with **1** and D_2. Signals for each C–D are given in the figure. The chemical shifts for $CH_3CH_2CD_2$=CDC(CH_3)P are from *J. Am. Chem. Soc.* **1998,** *120,* 2308-2321.

Representative high temperature NMR data of residual iPP after reactions with H_2 and $Cp_2HfMe_2/\equiv SiOAl(OC(CF_3)_3)_2(O(Si\equiv)_2)$.

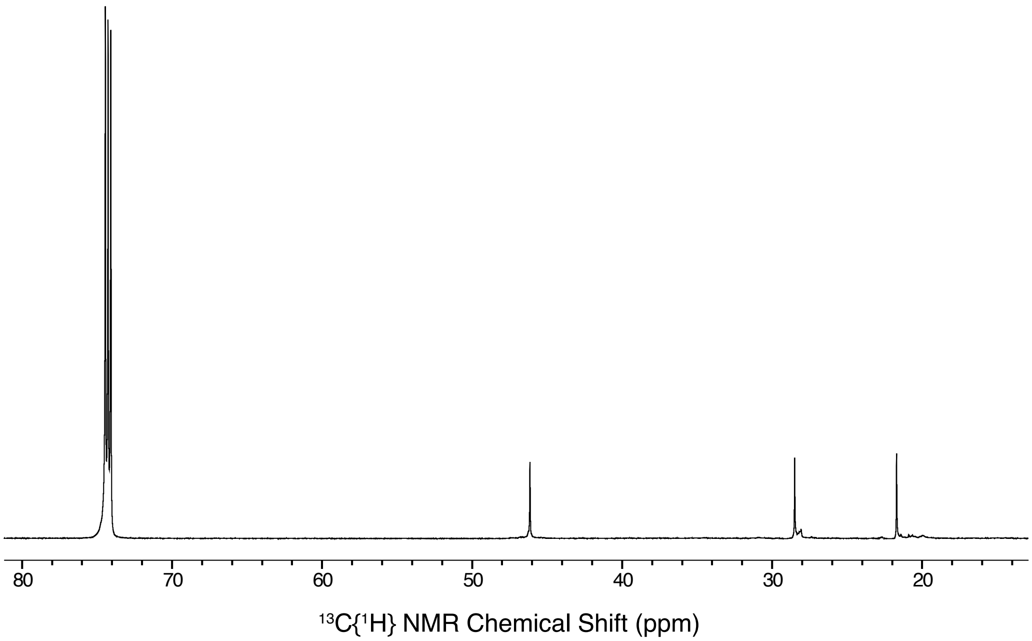

Figure S29. Quantitative $^{13}C\{^1H\}$ NMR spectrum of the residual polymer melt from the hydrogenolysis of iPP with $Cp_2HfMe_2/\equiv SiOAl(OC(CF_3)_3)_2(O(Si\equiv)_2)$ at 1atm, collected at 120˚C in $C_2D_2Cl_4$.

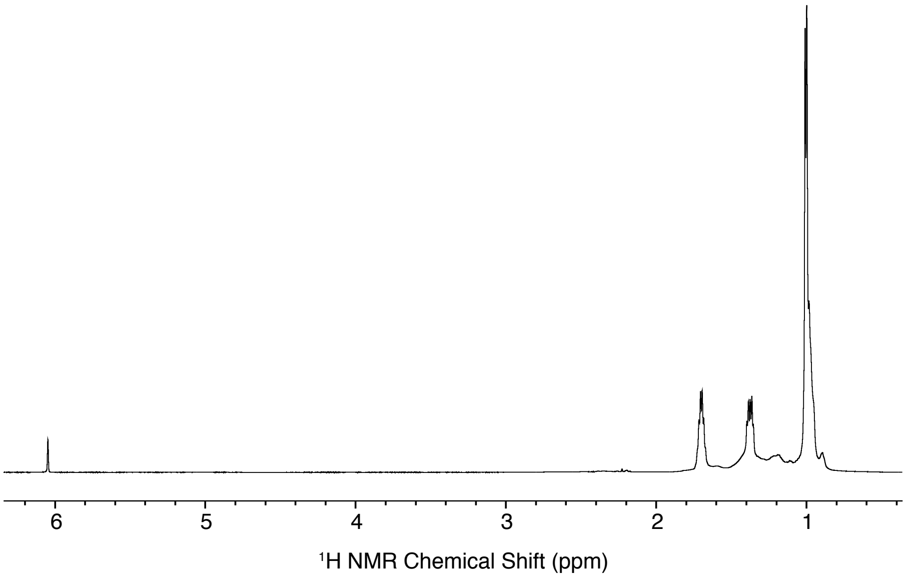

Figure S30. ^1H NMR spectrum of the residual polymer melt from the hydrogenolysis of iPP with $Cp_2HfMe_2/\equiv SiOAl(OC(CF_3)_3)_2(O(Si\equiv)_2)$ at 1atm, collected at 120˚C in $C_2D_2Cl_4$.

Table S2. Integral values obtained from quantitative $^{13}C\{^{1}H\}$ NMR data shown in Figure 4.

	Sum of Integrals for end groups	Sum of Integrals for all other carbons	Ratio of end group integral to C_3H_6 units	Calculated M_n[a] (g/mol)
1atm (H_2:Hf ~ 100)	1.46	35	8.1	388
1atm (H_2:Hf ~ 1500)	4.19	72.1	5.7	290
1atm (H_2:Zr ~ 1500)	3.74	79.3	7.06	347
5atm (Parr)	1	14.00	4.67	238
10atm (Parr)	1	12.84	4.28	221
2atm (on demand)	7.5	572	25.4	1136
5atm (on demand)	4.62	355.73	25.6	1146

[a] – All M_n values also include a propyl end group in the molecular weight.

iPP degradation reactions using ≡SiOAl(OC(CF_3)$_3$)$_2$(O(Si≡)$_2$).

In an argon-filled glovebox, a 100mL Schlenk flask fitted with a Teflon-tap was loaded with 200 mg iPP and 200 mg ≡SiOAl(OC(CF_3)$_3$)$_2$(O(Si≡)$_2$) (0.044 mmol Al). The flask was removed from the glovebox, connected to a high vacuum line, and evacuated for 5 min. The flask was filled with 1atm of H_2 (4.16 mmol), sealed, disconnected from the line, and heated at 200 ˚C for 24h. Volatiles were sampled directly from the flask and analyzed by GC FID. Following analysis of volatile gases, the flask was opened to ambient atmosphere to proceed with the extraction of oils and remaining solids. Dichloromethane (~10 mL) was added to the flask at room temperature, and the solution was decanted from the residual polymer melt and spent catalyst mixture. This was repeated two more times. The combined dichloromethane extract was concentrated by heating gently to remove the solvent. The yield of oil from this reaction was 30 mg (15 % from initial iPP mass).

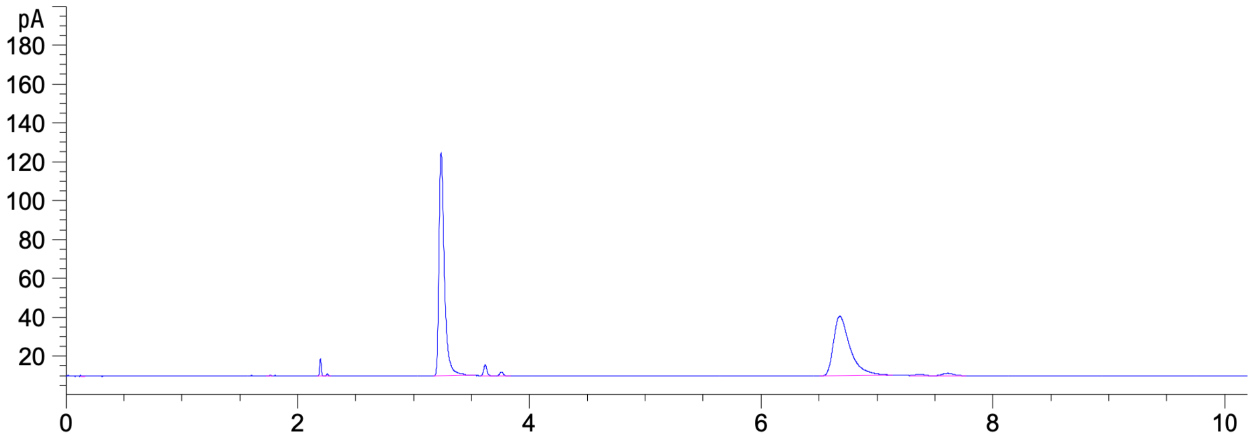

Figure S31. GC of the gas phase of iPP hydrogenolysis reactions with 200 mg ≡SiOAl(OC(CF_3)$_3$)$_2$(O(Si≡)$_2$). The amounts of gas evolved are 0 CH_4 Hf^{-1}, 0 C_2H_6 Zr^{-1}, 0.01 C_3H_6 Zr^{-1} and 0.4 C_4H_{10} Zr^{-1}, and 0.2 C_5H_{10} Zr^{-1}. The total yield of light gases are 0.01% (2 mg) based on this data.

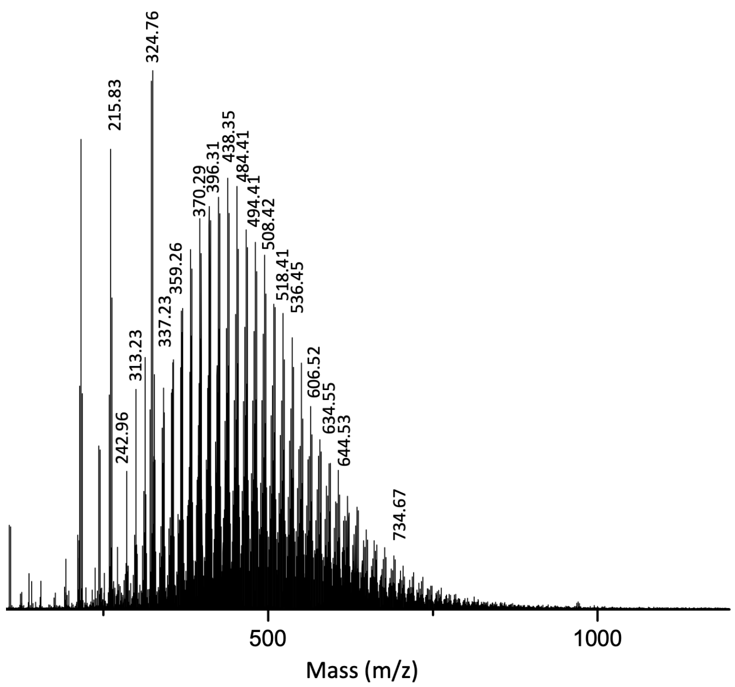

Figure S32. Representative MALDI MS of the oils using ≡SiOAl(OC(CF$_3$)$_3$)$_2$(O(Si≡)$_2$).

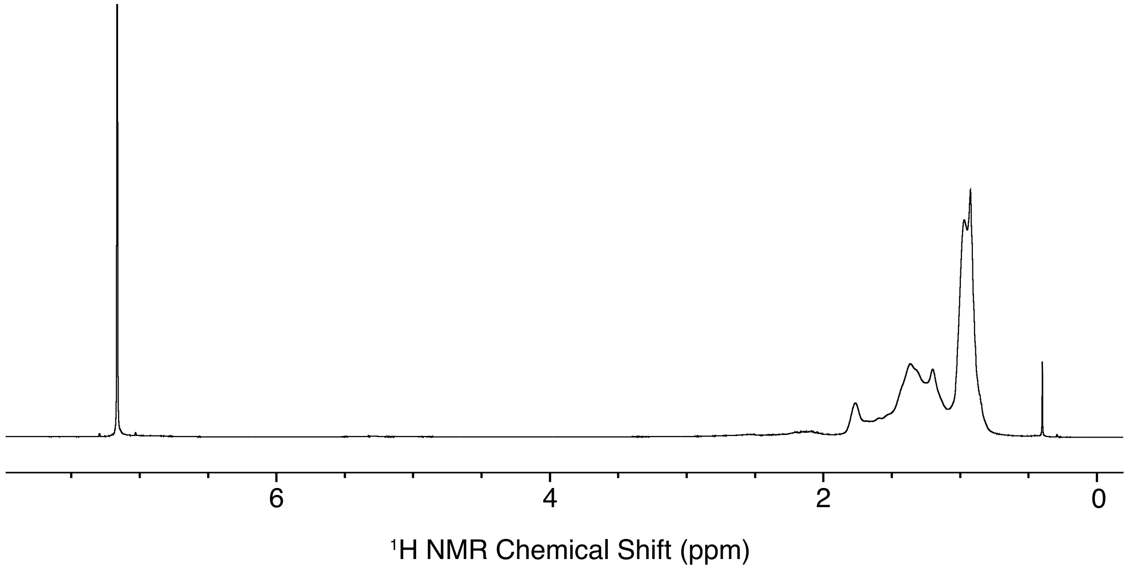

Figure S33. ^{1}H NMR of the oils using ≡SiOAl(OC(CF$_3$)$_3$)$_2$(O(Si≡)$_2$).

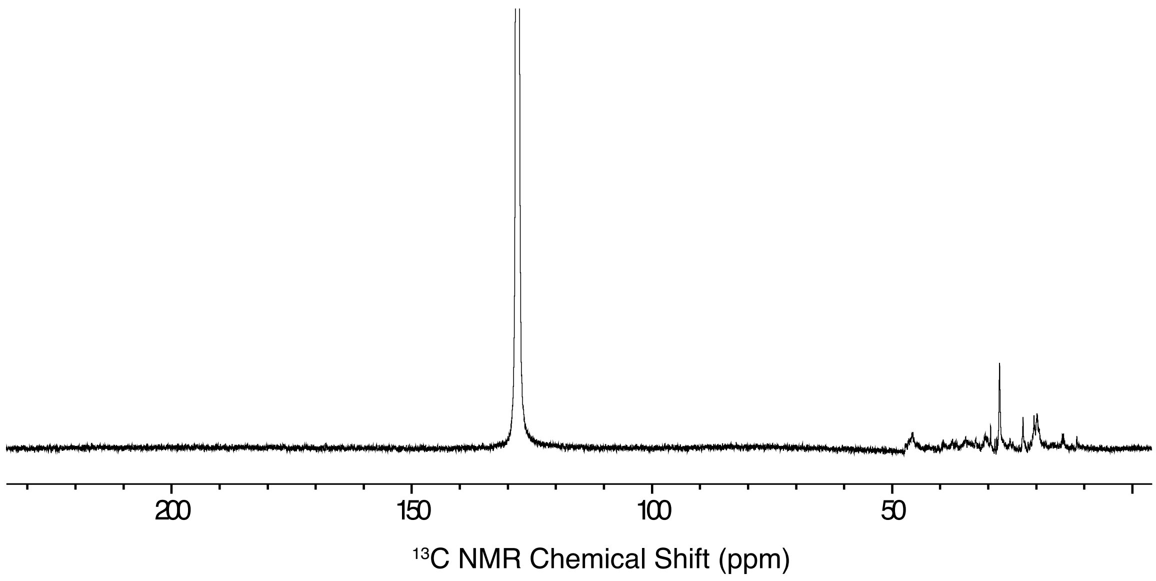

Figure S34. $^{13}C\{^{1}H\}$ NMR of the oils using $\equiv SiOAl(OC(CF_3)_3)_2(O(Si\equiv)_2)$.

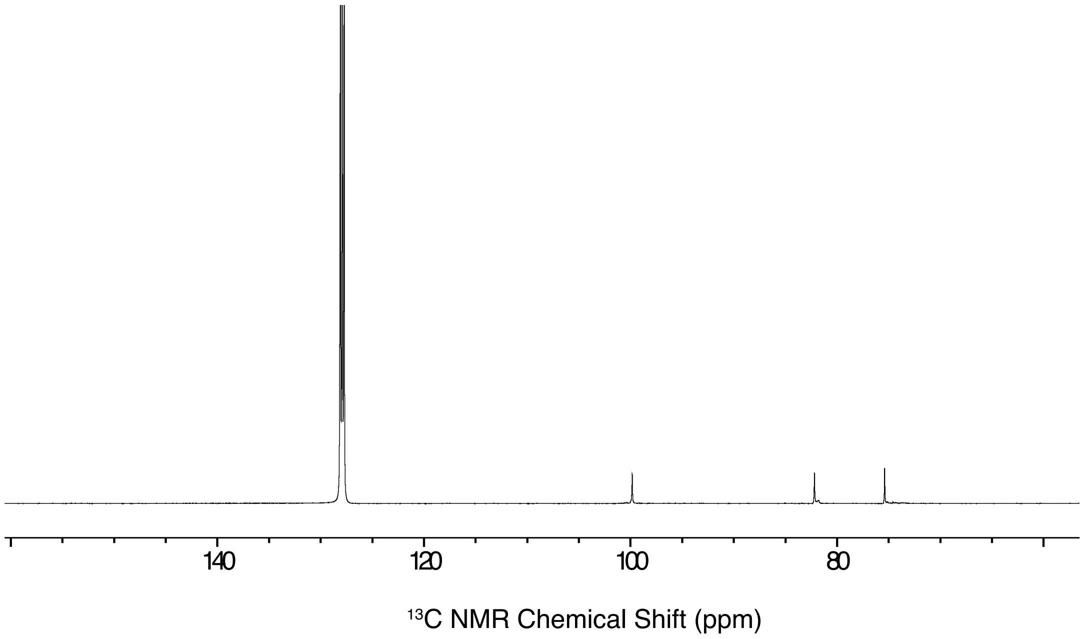

Figure S35. Quantitative $^{13}C\{^{1}H\}$ NMR of the residual iPP using $\equiv SiOAl(OC(CF_3)_3)_2(O(Si\equiv)_2)$ showing highly isotactic iPP remains after the reaction. Recorded at 120 °C in $C_2D_2Cl_4$

iPP degradation reactions using $Cp_2Hf(CH_3)(OSi\equiv)$ (2).

In an argon-filled glovebox, a 100mL Schlenk flask fitted with a Teflon-tap was loaded with 200 mg iPP and 200 mg $Cp_2Hf(CH_3)(OSi\equiv)$ (0.052 mmol Hf). The flask was removed from the glovebox, connected to a high vacuum line, and evacuated for 5 min. The flask was filled with 1atm of purified D_2 (4.16 mmol), sealed, disconnected from the line, and heated at 200 ˚C for 24h. Volatiles were sampled directly from the flask and analyzed by GC FID. Following analysis of volatile gases, the flask was opened to ambient atmosphere to proceed with the extraction of oils and remaining solids. Attempts to isolate oil using the procedures with other materials described here (successive dichloromethane (~10 mL) extracts), resulted in no isolable oil.

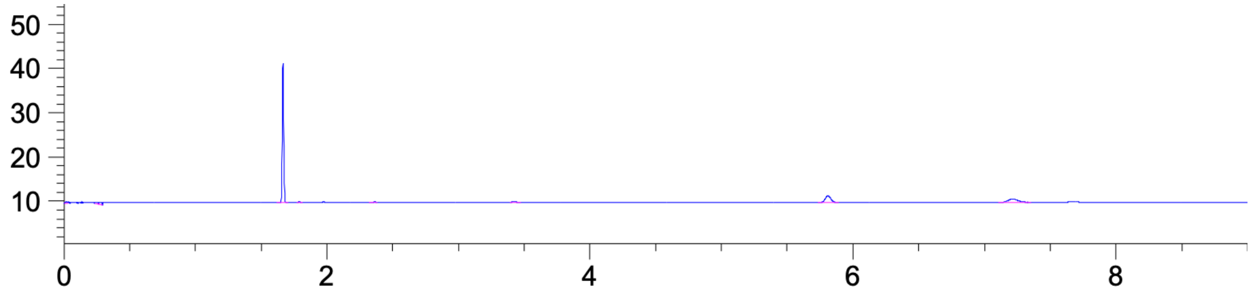

Figure S36. GC of the gas phase of iPP hydrogenolysis reactions with **200mg Hf/SiO2 1atm D2 control**. The amounts of gas evolved are 0.08 CH_4 Hf^{-1}, 0 C_2H_6 Zr^{-1}, 0 C_3H_6 Zr^{-1} and 0 C_4H_{10} Zr^{-1}, and 0.003 C_5H_{10} Zr^{-1}.

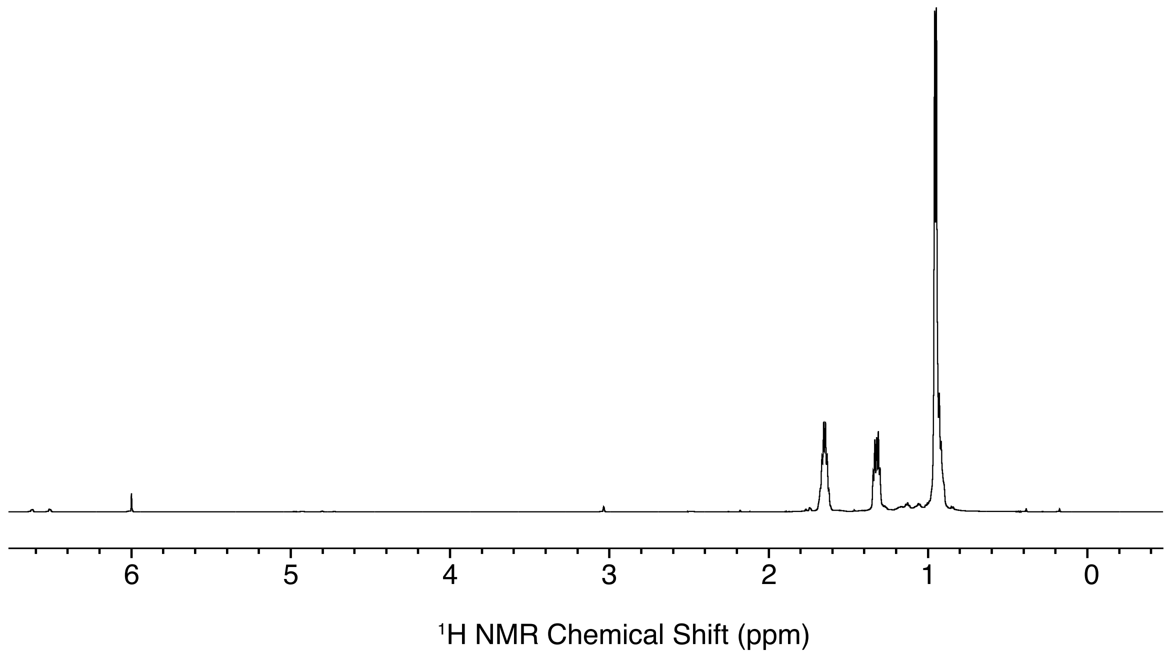

Figure S37. ^{1}H NMR of the residual iPP using **2**. Recorded at 120 °C in $C_2D_2Cl_4$.

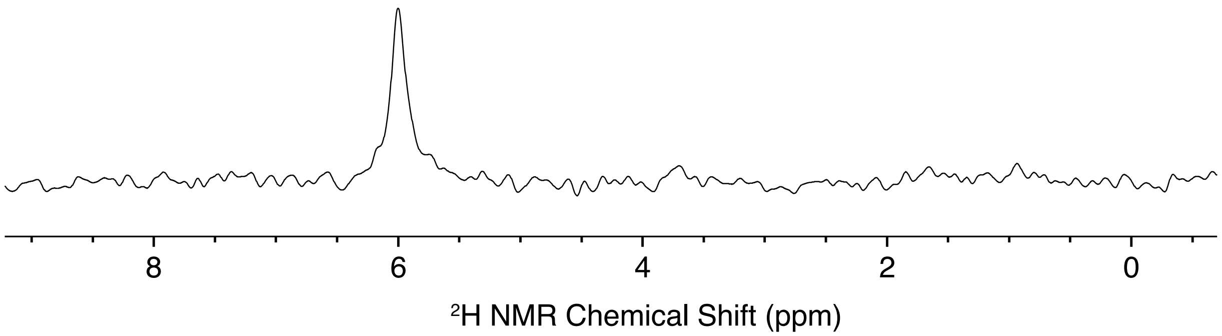

Figure S38. ^{2}H NMR of the residual iPP using **2**. Recorded at 120 °C in $C_2D_2Cl_4$. A ^{2}H NMR signal for any of the sites in iPP is not observed under these conditions (ns = 13590, d1 = 0.5 s, total experiment time = 6.5 h)

(1) Samudrala, K. K.; Huynh, W.; Dorn, R. W.; Rossini, A. J.; Conley, M. P. Formation of a Strong Heterogeneous Aluminum Lewis Acid on Silica. *Angew. Chemie - Int. Ed.* **2022**, *61* (40). https://doi.org/10.1002/anie.202205745.

(2) Jantunen, K. C.; Scott, B. L.; Kiplinger, J. L. A Comparative Study of the Reactivity of Zr(IV), Hf(IV) and Th(IV) Metallocene Complexes: Thorium Is Not a Group IV Metal after All. *J. Alloys Compd.* **2007**, *444–445* (SPEC. ISS.), 363–368. https://doi.org/10.1016/j.jallcom.2007.03.138.
